# Induction of Terpene Biosynthesis in Berries of Microvine Transformed with *VvDXS1* Alleles

**DOI:** 10.3389/fpls.2017.02244

**Published:** 2018-01-17

**Authors:** Lorenza Dalla Costa, Francesco Emanuelli, Massimiliano Trenti, Paula Moreno-Sanz, Silvia Lorenzi, Emanuela Coller, Sergio Moser, Davide Slaghenaufi, Alessandro Cestaro, Roberto Larcher, Ivana Gribaudo, Laura Costantini, Mickael Malnoy, M. Stella Grando

**Affiliations:** ^1^Research and Innovation Centre, Fondazione Edmund Mach, Genomics and Biology of Fruit Crop Department, San Michele all'Adige, Italy; ^2^Center Agriculture Food Environment, University of Trento, San Michele all'Adige, Italy; ^3^Research and Innovation Centre, Fondazione Edmund Mach, Computational Biology Platform, San Michele all'Adige, Italy; ^4^Technology Transfer Centre, Fondazione Edmund Mach, Experiment and Technological Services Department, San Michele all'Adige, Italy; ^5^Institute for Sustainable Plant Protection—CNR, Grugliasco, Italy

**Keywords:** functional SNP, gain-of-function mutation, microvine, monoterpene, sesquiterpene, TaqMan card, *Vitis vinifera*, *VvDSX1* alleles

## Abstract

Terpenoids, especially monoterpenes, are major aroma-impact compounds in grape and wine. Previous studies highlighted a key regulatory role for grapevine 1-deoxy-D-xylulose 5-phosphate synthase 1 (VvDXS1), the first enzyme of the methylerythritol phosphate pathway for isoprenoid precursor biosynthesis. Here, the parallel analysis of *VvDXS1* genotype and terpene concentration in a germplasm collection demonstrated that *VvDXS1* sequence has a very high predictive value for the accumulation of monoterpenes and also has an influence on sesquiterpene levels. A metabolic engineering approach was applied by expressing distinct *VvDXS1* alleles in the grapevine model system “microvine” and assessing the effects on downstream pathways at transcriptional and metabolic level in different organs and fruit developmental stages. The underlying goal was to investigate two potential perturbation mechanisms, the former based on a significant over-expression of the wild-type (neutral) *VvDXS1* allele and the latter on the *ex-novo* expression of an enzyme with increased catalytic efficiency from the mutated (muscat) *VvDXS1* allele. The integration of the two *VvDXS1* alleles in distinct microvine lines was found to alter the expression of several terpenoid biosynthetic genes, as assayed through an *ad hoc* developed TaqMan array based on cDNA libraries of four aromatic cultivars. In particular, enhanced transcription of monoterpene, sesquiterpene and carotenoid pathway genes was observed. The accumulation of monoterpenes in ripe berries was higher in the transformed microvines compared to control plants. This effect is predominantly attributed to the improved activity of the VvDXS1 enzyme coded by the muscat allele, whereas the up-regulation of *VvDXS1* plays a secondary role in the increase of monoterpenes.

## Introduction

Isoprenoids, also known as terpenoids, are the largest family of plant natural compounds with many biological functions including growth and development (gibberellic acid, abscisic acid, brassinosteroids, and cytokinins), photosynthesis (chlorophylls, carotenoids, plastoquinones), defense as well as interaction with the environment (monoterpenes, sesquiterpenes, and diterpenes) (Dudareva et al., 2004; Aharoni et al., 2005; Tholl, 2006). From a human perspective, isoprenoids are also of commercial interest (Aharoni et al., 2005). Some of them play a direct role in the fruit quality, such as the monoterpenes linalool, geraniol, nerol that are the major aromatic determinants in Muscat grape varieties, others are used as flavors and fragrances in foods and cosmetics (e.g., menthol, nootkatone, and sclareol) or for medical applications (e.g., taxol, artemisinin, and glycyrrhizin). Isoprenoids derive from both the mevalonate pathway (MVA), which is active in the cytosol, and the plastidial 2-C-methyl-D-erythritol-4-phosphate (MEP) pathway through the formation of the common precursor intermediates isopentenyl diphosphate (IPP) and dimethylallyl diphosphate (DMAPP) (Laule et al., 2003). The MVA route is responsible for the formation of sesquiterpenes, triterpenes, sterols, and the prenyl chain of ubiquinone, while the MEP pathway is involved in the biosynthesis of isoprene, monoterpenes, diterpenes, carotenoids, the phytyl side chain of chlorophyll and the prenyl chain of plastoquinone (Eisenreich et al., 2004). In spite of this compartmentalization, a crosstalk between the cytosolic and plastidial pathways has been demonstrated in Arabidopsis, which takes place preferentially from the chloroplast to the cytoplasm (Laule et al., 2003; Dudareva et al., 2013).

The ecological and commercial importance of terpenoids makes their manipulation through metabolic engineering an attractive challenge, as proved by intensive research in recent years (Farhi et al., 2011; Dong et al., 2013; Houshyani et al., 2013; Lange and Ahkami, 2013). Although highly appealing from a biotechnological viewpoint, this goal is not easy to achieve (McCaskill and Croteau, 1997). Metabolic pathways are controlled at multiple levels and any form of perturbation can have wide-ranging effects at the whole system level (Capell and Christou, 2004). Therefore, the modulation of key regulatory enzymes may result in an altered production of various metabolites.

Studies from the model plant *Arabidopsis thaliana* (Mandel et al., 1996; Estévez et al., 2000, 2001) suggested that the control of the MEP pathway flux is primarily exerted by the first enzyme of the route, 1-deoxy-D-xylulose 5-phosphate synthase (DXS). The key role of DXS in the plastidial isoprenoid biosynthesis was subsequently proved in other plant species, including *Lycopersicon esculentum* (Lois et al., 2000; Enfissi et al., 2005), *Whitania somnifera* (Jadaun et al., 2017), *Solanum tuberosum* (Morris et al., 2006), *Lavandula latifolia* (Muñoz-Bertomeu et al., 2006), *Catharanthus roseus* (Peebles et al., 2011), *Daucus carota* (Simpson et al., 2016), *Salvia sclarea* (Vaccaro et al., 2014), *Salvia milthiorrhiza* (Zhou et al., 2016). It was further demonstrated that the MEP pathway is controlled by tight feedback regulation of the reaction catalyzed by DXS (Wolfertz et al., 2004; Flores-Pérez et al., 2008; Wright et al., 2014).

In several plant species DXS is encoded by more than a single gene and each isoform displays differential expression during development and in specific organs, suggesting a non-redundant function (Rodríguez-Concepción and Boronat, 2002; Khemvong and Suvachittanont, 2005; Kim et al., 2005; Phillips et al., 2007; Cordoba et al., 2011; Han et al., 2013; Saladié et al., 2014; Xu et al., 2014).

Multiple *DXS* gene isoforms were also predicted in grapevine, where *VvDXS1* is located on chromosome 5, three *VvDXS2* isoforms (*VvDXS2A, VvDXS2B*, and *VvDXS2C*) located on chromosomes 15, 11, and 7 respectively, and *VvDXS3* on chromosome 4 (Battilana et al., 2009). Over the last few years, *VvDXS1* was discovered to co-localize with the major QTL (quantitative trait locus) for monoterpene content in mature grape berry (Battilana et al., 2009) and a non-neutral dominant mutation in this gene causing an amino acid exchange from K (Lysine) to N (Asparagine) at position 284 of the protein was found to be significantly associated with muscat-flavored grapevine varieties (Emanuelli et al., 2010). This mutation was shown to improve the enzymatic catalytic efficiency *in vitro* and to cause a dramatic increase of glycosylated monoterpenes in transgenic tobacco overexpressing the *VvDXS1* N284 allele (Battilana et al., 2011).

In the present study the functional analysis of *VvDXS1* was carried out for the first time in grapevine using the microvine model system (Chaïb et al., 2010) modified to ectopically express either the mutated (N284) or the non-mutated (K284) form of the gene. A TaqMan array tool was developed in order to simultaneously evaluate the expression pattern of a hundred terpenoid biosynthetic genes in transformed microvines at various stages during berry development. This allowed to investigate how a potentially enhanced MEP pathway flux may perturb isoprenoid metabolism. In addition, the content of free and bound monoterpenes, as well as sesquiterpenes, was assessed in mature berries. The results are discussed with reference to natural variation of *VvDXS1* and terpene concentration in the grapevine (*Vitis vinifera* L.) germplasm.

## Materials and methods

### Plant material and gene transfer

*Agrobacterium tumefaciens* (*A.t*.)-mediated gene transfer was performed on embryogenic calli of “microvine 04C023V0006” (derived from a cross between “Grenache” and the original L1 mutant microvine, Chaïb et al., 2010), “Chardonnay” and “Brachetto” genotypes according to Dalla Costa et al. (2014). Experiments were carried out using *A.t*. strain EHA105 (Hood et al., 1993) carrying a pK7WG2 plant binary vector (Karimi et al., 2002), with the muscat (M) or the neutral (N) allele of *VvDXS1* under the control of the CaMV-35S promoter. The two forms of the gene differ for one nucleotide substitution at position 1822 (G in the neutral allele or T in the muscat allele), which results in the substitution of Lysine with Asparagine at position 284 in the protein sequence. As selectable marker the neomycin phosphotransferase II (*nptII*) gene was used, which confers resistance to kanamycin.

Transformed and wild-type *in vitro* plantlets were acclimatized in a growth chamber (94.5 μmol·m^−2^·s^−1^ cool white light and 16 h-light photoperiod, at 25°C and 70% humidity) and subsequently transferred to the greenhouse.

### Allele discrimination by *VvDXS1* amplicon digestion and sequencing

The PCR was performed in a 20 μl reaction volume containing 100 ng of leaf DNA, 0.25 mM dNTPs, 0.3 μM of each primer (Fw: ATTGCTGTCATAGGTGATGGAG; Rv: CTGTTGTCTTGGTACTCTTAAC), 1X Taq Buffer Advanced (5 Prime, Hilden, Germany) and 1 unit of 5 Prime Taq DNA Polymerase (5 Prime, Hilden, Germany). The initial denaturation at 95°C for 5 min was followed by 35 cycles of 30 s at 95°C, 30 s at 58°C, and 30 s at 68°C, with a final extension of 7 min at 68°C. The obtained amplicon (423 bp) was digested with the FastDigest *StyI* restriction enzyme (Thermo Fischer Scientific, Waltham, MA, USA) to detect SNP1822 G/T (*StyI* recognizes the restriction site when G is present) or sequenced with the 3730xl DNA Analyzer (Applied Biosystems, Foster City, CA, USA).

### Estimation of transgene insertion copies through southern blot and qPCR

Digoxigenin-labeled probes for *VvDXS1* and *nptII* genes were obtained with the PCR Dig Probe Synthesis Kit (Roche Diagnostics, Indianapolis, IN, USA), using the following primers: *VvDXS1*-SB-fw = ATGGCTCTCTGTACGCTCTCA and *VvDXS1*-SB-rv = AGTTGTTTCAGCTCCTTGACAG; *nptII*-SB-fw = GAAGGGACTGGCTGCTATTG and *nptII*-SB-rv = AATATCACGGGTAGCCAACG. For each sample, 10 μg of DNA was digested with the Fastdigest® restriction enzymes *XbaI* and *EcoRI* (Thermo Fischer Scientific, Waltham, MA, USA) for *VvDXS1* probing and with *HindIII* for *nptII* probing. Digestion products were precipitated, resuspended in 30 μl Milli-Q water and separated overnight on 0.9% agarose gel (0.5X TBE) at 50 V. Membrane blotting and hybridization were performed following Roche user's manual. The autoradiographic film was exposed overnight before development.

Quantitative real-time PCR (qPCR) amplification was performed on genomic DNA in 96-well reaction plates on the iCycler iQ Thermocycler (Biorad, Hercules, CA, USA) according to the method by Dalla Costa et al. (2009).

### Gene expression analysis

Total RNA was isolated from grape leaves, flowers and fruits at different development stages using the Spectrum™ Plant Total RNA Kit (Sigma Aldrich, St. Louis, MO, USA) and quantified with the spectrophotometer NanoDrop ND-8000 (NanoDrop Technologies, Wilmington, DE, USA). Following DNase treatment, 2 μg of RNA were retro-transcribed into cDNA with the SuperScript® III Reverse Transcriptase (Invitrogen, Carlsbad, CA, USA) and oligo(dT) (or random primers for applications with TaqMan array cards). The qPCR was carried out as described in Supplementary Materials.

### TaqMAN array card development and assay

A set of terpenoid biosynthetic genes were selected for loading onto the TaqMan array card based on their expression in aromatic grapevine cultivars. For this purpose, four normalized cDNA libraries were obtained using the extracted RNA from ripening berries of the cultivars Gewürztraminer (TRA), Malvasia di Candia aromatica (MAL), Moscato Bianco (MOB) and Rhein Riesling (RIE). The normalized cDNA libraries were then sequenced with a 454 GS FLX Titanium system (Roche, Indianapolis, IN, USA) and filtered high quality reads were aligned to the 12x V1 version of the *Vitis vinifera* genome (details are provided in Supplementary Materials). In parallel, a total of 180 genes involved in terpenoid biosynthesis in grapevine were retrieved from KEGG (http://www.genome.jp/kegg-bin/show_organism?org=vvi) by searching for the following ko terms: vvi00900 (terpenoid backbone biosynthesis), vvi00902 (monoterpenoid biosynthesis), vvi00904 (diterpenoid biosynthesis), vvi00905 (brassinosteroid biosynthesis), vvi00906 (carotenoid biosynthesis) and vvi00909 (sesquiterpenoid and triterpenoid biosynthesis). An additional 20 genes were selected based on their co-localization with grapevine QTLs for monoterpenoid content (Battilana et al., 2009) or peculiar expression in ripening berries of aromatic grapes (unpublished data). All the above mentioned genes were checked for their expression in the normalized cDNA libraries and were manually investigated in order to precisely define their gene structure, allelic and possible splicing variants.

TaqMan array (TA) cards (Applied Biosystems, Foster City, CA, USA) are 384-well microfluidic cards with eight ports, each containing 48 connected wells. Primers and probes are preloaded and dried onto the wells by the manufacturer at the following concentrations: 9 × 10^−7^ mol/L for primer, 2 × 10^−7^ mol/L for probe. All the probes are conjugated at 5′ to a reporter 6-carboxyfluorescein (FAM) and at 3′ to a non-fluorescent quencher (NFQ) with the minor groove binder (MGB) moiety attached to the molecule. In the TaqMan array card developed in our study (Aromix), four samples (for details see Results) can be assessed simultaneously for 83 targets connected with the terpene metabolism (Table S1). The card also features five endogenous genes, as well as the manufacturer's card control PCR. Probes and primers were designed by the manufacturer based on the gene sequences supplied by the authors. All TA cards were run on the ViiA™ 7 Real-Time PCR System (Applied Biosystems, Foster City, CA, USA). Each of the eight ports of the card was loaded with a 100 μl solution obtained by mixing 50 μl of 1:20 diluted cDNA with 50 μl of TaqMan Gene Expression Master Mix 2X (Applied Biosystems, Foster City, CA, USA). The cards were centrifuged twice at 1,200 rpm for 1 min and sealed, the loading ports were excised and the cards were placed in the thermal cycler. The following cycling conditions were used: 50°C for 2 min, 95°C for 10 min, and 40 cycles of 95°C for 30 s followed by 60°C for 1 min. Results were processed with qbase^PLUS^ software (Biogazelle, Zwijnaarde, Belgium; Hellemans et al., 2007) and normalized by the reference genes selected from qbase^PLUS^ (actin and glyceraldehyde-3-phosphate dehydrogenase).

### Terpenoid quantification in grapevine germplasm and in microvine plants transformed with *VvDXS1*

Approximately 90 grapevine cultivars representing Muscat, herbaceous, or other distinct flavored cultivars, as well as non-aromatic ones were analyzed at technological maturity (harvest time) for monoterpene and sesquiterpene content in whole berries. Berry flavor phenotype as assessed merely by tasting and the *VvDXS1* genotype were previously reported for the majority of these accessions (Emanuelli et al., 2014).

For each microvine line, berry number and size, total leaf area and photosynthetic activity were evaluated in four biological replicates 1 month before berry sampling. Targeted cluster thinning or leaf pruning were carried out in order to set a similar ratio between total leaf area and number of berries for all the plants under analysis (Table S2). Clusters were collected at technological maturity (18–20.5 degrees Brix,°Bx) and berry skin was separated from the pulp. Skins were grounded to a fine powder in liquid nitrogen and used for monoterpene and sesquiterpene analysis.

Eleven monoterpenes were extracted from the starting plant material and quantified in their free and glycosidically bound form by solid phase extraction (SPE) and high-resolution gas chromatography-mass spectrometry (HRGC-MS), as previously described by Battilana et al. (2011). A non-targeted analysis was carried out to profile the sesquiterpenes.

## Results

### Characterization of transformed plants

**Figure 1:**
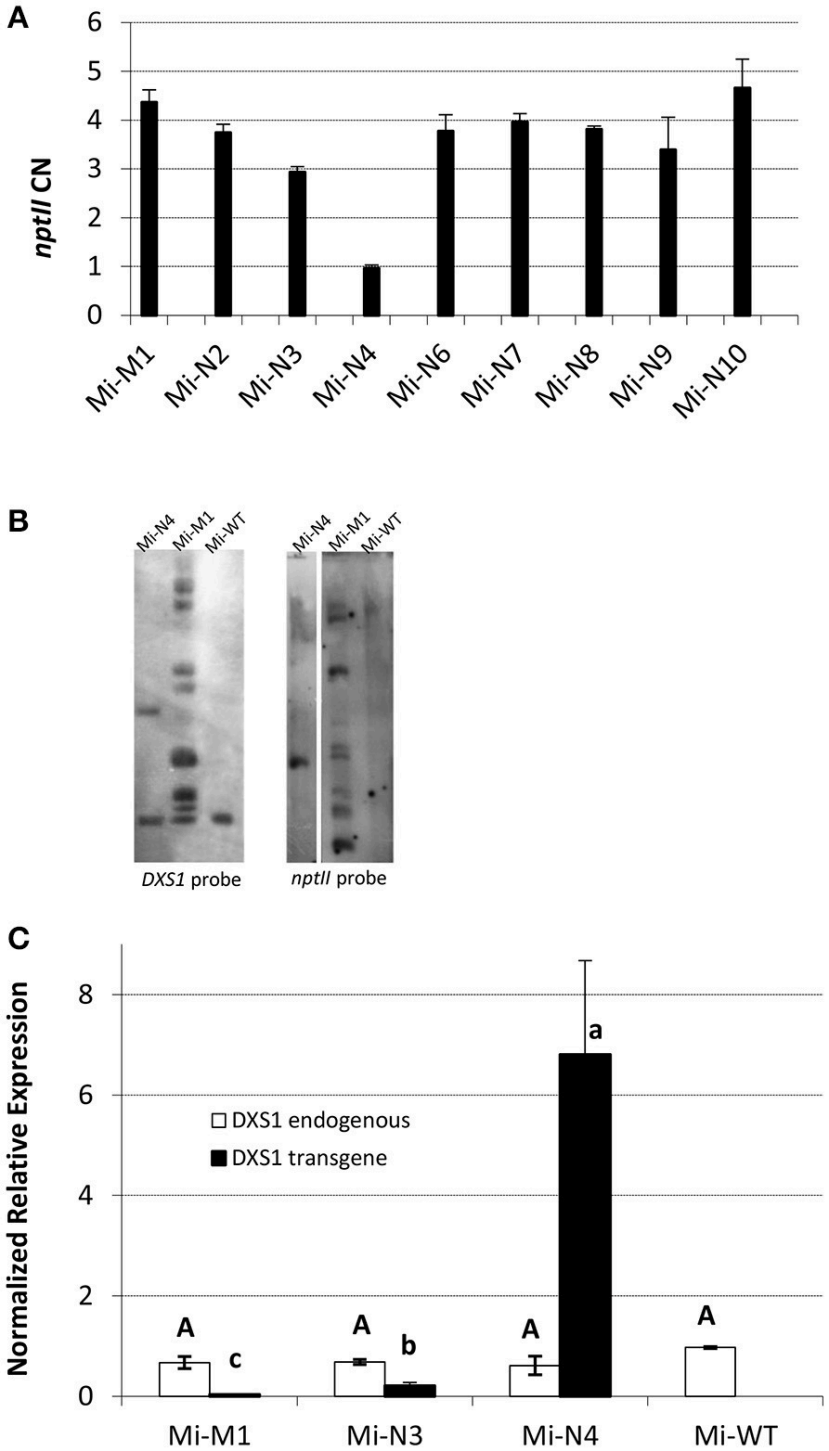
Molecular characterization of *in vitro* transformed microvines. **(A,B)** Determination of T-DNA integration copies by real-time PCR for *nptII* quantification (copy number (CN) values are the mean ± standard error (SE) of two biological replicates analyzed in two separate PCR sessions) **(A)** and by Southern blot with *VvDXS1* and *nptII* probes **(B)**. **(C)** Transcription profile of endogenous (white) and transgenic (black) *VvDXS1* in the leaf tissue. Expression values are the mean ± SE of three biological replicates. Uppercase and lowercase lettering on the bars indicate different subsets according to ANOVA and Tukey's HSD *post-hoc* tests (*P* < 0.05). WT, wild-type; M, *VvDSX1* muscat allele; N, *VvDXS1* neutral allele.

Genetically modified plants were obtained from microvine (Mi), Chardonnay (C) and Brachetto (B) genotypes (Figure 1, Figure S1). Microvines were chosen, since they have short generation cycles and continuous flowering, which ensure fruit production and significantly reduce the time required for genetic studies in grapevine (Chaïb et al., 2010). The identity of the *VvDXS1* integrated allele (neutral or muscat form) was confirmed by sequencing. The number of T-DNA integration copies as calculated by real-time PCR and Southern blot is shown in Figures 1A,B, Figure S1A. One T-DNA integration copy was detected for Mi-N4, two for C-M5, C-M6 and B-N8, while Mi-M1 and the remaining lines transformed with the neutral allele presented a multi-copy insertion.

**Figure 2:**
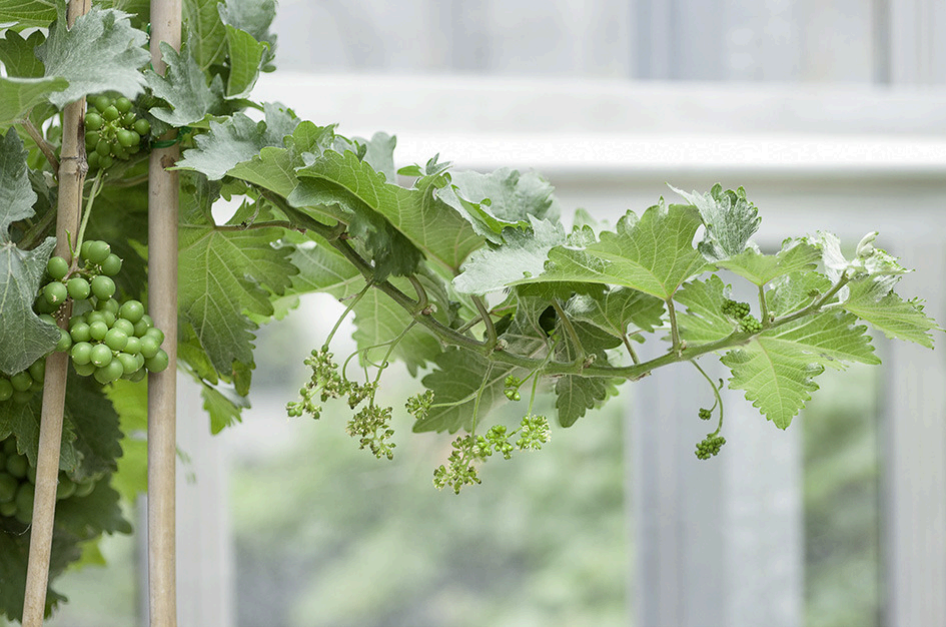
A shoot of microvine 04C023V0006 with flowers and berries at different developmental stages.

The qPCR expression analysis proved that *VvDXS1* transgene was transcribed in the leaves of all transformed lines, although the level of expression was variable across the lines. Conversely, endogenous *VvDXS1* was stably expressed (Figure 1C, Figures S1B, S2). After this evaluation, Chardonnay and Brachetto plants—which were not expected to produce fruits at least in the short-term—were not further investigated and were kept for future experiments. Several biological replicates of each transformed and WT microvine line were transferred to soil and successfully acclimatized in the greenhouse in 2013, 2014, and 2015 (Figure 2). Data on flowering time and berry development were collected for Mi-M1, Mi-N3, Mi-N4, and Mi-WT acclimatized in January 2014 (Table 1). The first microvine plants began to bloom about 150 days after acclimatization and anthesis occurred on average 197 days after acclimatization in 11 out of the 14 observed plants. Eight microvine plants reached *veraison* and maturity, respectively 281 and 309 days (on average) after acclimatization. Modified plants did not show any evident phenotypic difference *in vitro* or in greenhouse conditions, compared to the control plants (Table S2).

**Table 1:** Time required to reach anthesis, *veraison* and maturity starting from acclimatization, for three transformed microvine lines and control microvine (WT).

**Acclimatized microvine lines**	**Anthesis**		***Veraison***		**Maturity**	
	Days^*^	**Range**	Days^*^	**Range**	Days^*^	**Range**
Mi-M1 (5)	214 (4)	172–234	279 (4)	238–298	304 (4)	267–323
Mi-N3 (3)	181 (2)	144–218	-		-	
Mi-N4 (3)	187 (3)	145–214	277 (2)	246–308	303 (2)	274–331
Mi-WT (3)	204 (2)	198–210	286 (2)	282–289	320 (2)	316–323
Average	197		281		309	

The number of biological replicates of each line is reported in brackets. Anthesis was considered as the moment when 50% of flowers were fully open and veraison when 50% of berries changed color and softened.

* *Mean value of the biological replicates available for each line*.

### *VvDXS1* expression in various tissues and developmental stages

**Figure 3:**
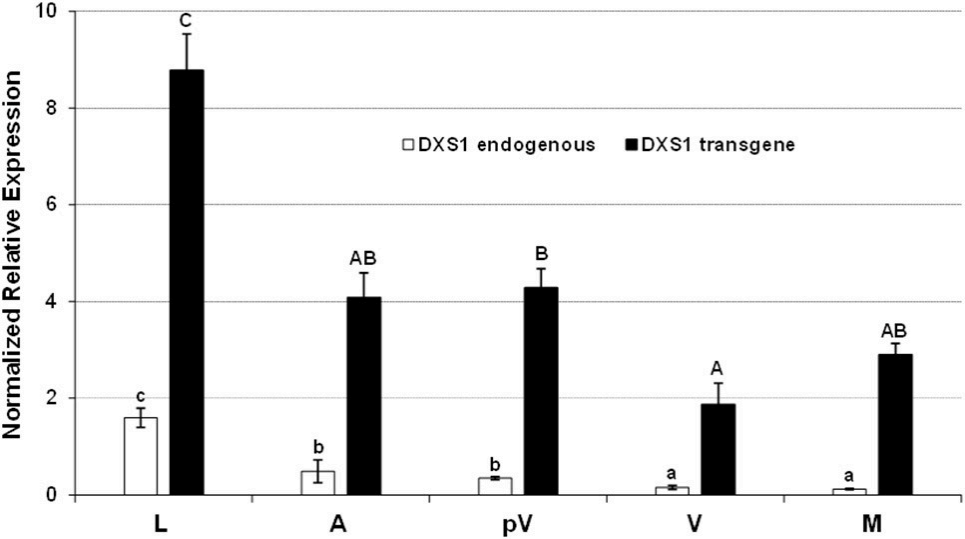
Expression analysis of endogenous (white) and transgenic (black) *VvDXS1* in different organs and berry developmental stages of the line Mi-N4. The same cDNAs employed for the TaqMan card assay were assessed from anthesis onwards. Expression values are the mean ± SE of two biological replicates analyzed in two separate PCR sessions (in the case of leaves, four biological replicates were considered). Uppercase and lowercase lettering on the bars indicate different subsets according to ANOVA and Tukey's HSD *post-hoc* tests (*P* < 0.05). L, leaves; A, flowers at anthesis; pV, berries at pre-*veraison* stage; V, berries at *veraison* stage; M, berries at technological maturity (18 °Brix).

The expression of both endogenous and transgenic *VvDXS1* in different plant tissues and during fruit development was investigated in Mi-N4, the microvine line with the highest transcription of the single copy transgene in leaves, and in Mi-M1, the only microvine line transformed with the muscat allele (Figure 1). The mRNA level of *VvDXS1* transgene was significantly higher in the leaf in comparison to flower at anthesis (stage E-L 21 of the modified E-L system by Coombe, 1995) and berry at pre-*veraison* (E-L 32), *veraison* (E-L 35) and maturity (E-L 38). Following berry development, the transgene transcript level decreased from pre-*veraison* to *veraison* and returned to increase after *veraison*. A similar trend was observed for the endogenous *VvDXS1* mRNA, even though the recovery following *veraison* was not detected (Figure 3, Figure S3). A comparable time-course profile, with the minimum point of the convexity corresponding to the *veraison* stage, was also obtained from the total *VvDXS1* expression analysis in three microvine lines (Figure S4).

### Development of a tool to quantify the transcriptional profile of terpenoid genes in grapevine (Aromix_Taqman array card)

In order to define a minimal gene set that could describe the transcriptional perturbation of terpenoid biosynthesis, a manual selection of candidate genes actually expressed during berry ripening in four aromatic grapevine cultivars was performed. By combining the cDNA libraries of the four varieties a total of 21246 genes was found to be expressed (17603, 18032, 17724, and 18198 in MAL, MOB, RIE, and TRA libraries, respectively), of which 14875 were common to all four cultivars (data not shown).

Two-hundred genes involved in terpenoid biosynthesis were checked for their expression in the normalized cDNA libraries and a final set of 78 genes (including five endogenous genes) was selected to be included in the TaqMan array card. For 10 of these genes two specific probes were designed in order to discriminate between splicing variants, which resulted in a total of 88 probes (Table S1).

The relative expression of these genes was evaluated in Mi-M1, Mi-N4, and Mi-WT plants during the 2014 growing season at four phenological stages (Table S3). In addition to the five endogenous genes, 64 terpenoid biosynthetic genes were expressed in the plants under study, of which 47 were expressed in all the investigated phenological stages while 17 were expressed only in some stages. Finally, nine genes on the TaqMan array could not be amplified, the majority of which (six) coded for terpene synthases.

**Figure 4:**
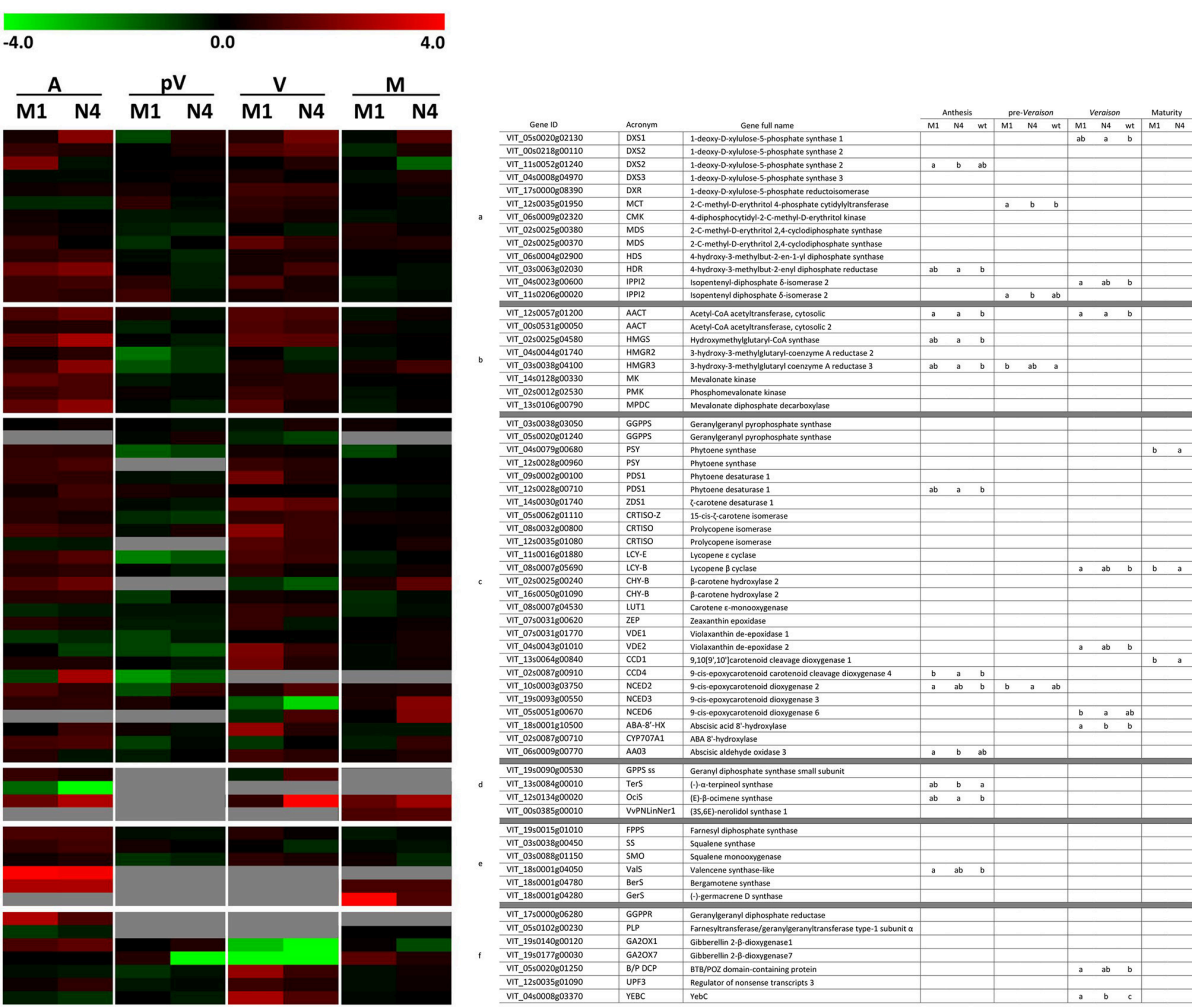
Relative expression matrix of the terpenoid pathway genes assayed by the Aromix_TaqMan array card in transformed and WT microvines during fruit development. Columns represent the transformed lines (M1 = Mi-M1, N4 = Mi-N4) at different stages (A, anthesis; pV, pre-*veraison*; V, *veraison*; M, maturity), while rows represent the genes grouped into six blocks related to terpenoid pathways: (a) MEP, (b) MVA, (c) carotenoids/apocarotenoids, (d) monoterpenes, (e) sesquiterpenes and (f) others (chlorophyll, gibberellin, isoprene, tocopherol, etc). Each element of the matrix indicates the log_2_ expression fold change in Mi-M1 or Mi-N4 lines compared to WT by means of a color code (heatmap). To this purpose, the raw Cq values of each line were considered stage by stage. The associated relative expression, which is the mean of two biological replicates both analyzed in duplicate, was calculated with the qBasePLUS software and is reported in Table S3 (at maturity only one WT biological replicate was available). Gray spaces correspond to not detectable transcripts. The heatmaps were generated with TM4 Multi experiment viewer (MeV) software (Saeed et al., 2003). The letters in the last four columns indicate different subsets according to ANOVA and LSD tests (anthesis, pre-*veraison, veraison*) and *t*-test (maturity) (*P* < 0.05).

Overall, the genes belonging to the MEP and the MVA pathways showed an evident up-regulation in the transformed lines at anthesis and *veraison* and a moderate down-regulation in the pre-*veraison* stage compared to WT. This general trend was also observed for the genes involved in the metabolism of carotenoids while the monoterpene and sesquiterpene biosynthetic genes were more highly expressed at anthesis. Based on ANOVA and *t*-tests, 24 genes showed significantly different expression (*P* < 0.05) among the analyzed plants during the four phenological stages (12 at anthesis, nine at *veraison*, four at pre-*veraison* and three at maturity) (Figure 4).

The simultaneous analysis of splicing variants of ten genes did not highlight relevant differences in most cases. However, for VIT_04s0079g00680 (*PSY*) and VIT_12s0057g01200 (*AACT*) only one splicing variant was expressed throughout berry development. A differential profile was also observed when both isoforms were expressed at least in one stage, as for VIT_06s0009g00770 (*AAO3*) and VIT_18s0001g10500 (*ABA 8*′*-HX*) (Figure S5, Table S3).

The expression data derived from the TaqMan card assay were validated with sybr-green real-time PCR on a set of relevant genes belonging to the MEP-, MVA-, carotenoid-, sesquiterpene-, and monoterpene biosynthetic pathways (Table S4). A strong correlation between the two sets of measurements was observed, with Pearson correlation coefficient (R) values approximately or >0.9 for seven genes and between 0.8 and 0.7 for three genes (Figure S4). Difference in expression levels between transformed and WT lines was the most notable at anthesis, and therefore a further expression analysis on a set of modulated genes (*VvDXS1, VvAACT, VvHMGS, VvHMGR3, VvOciS, VvFPPS, VvValS*) was repeated by qPCR on flowers collected in triplicate in 2016. The significant differences found in 2014 were confirmed in 2016 (data not shown).

### Effect of the *VvDXS1* mutation on the accumulation of terpenoids

#### Terpenoid content in the grapevine germplasm

**Figure 5:**
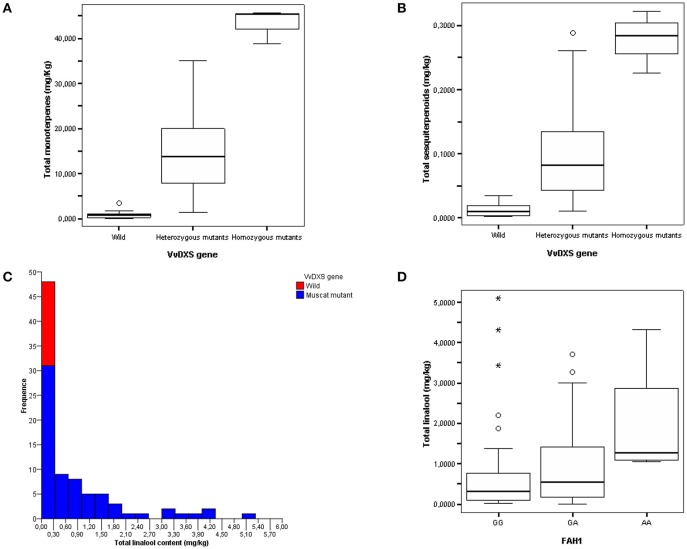
Total monoterpene **(A)** and sesquiterpene **(B)** content in the FEM aromatic core collection classified according to the *VvDXS1* genotype at position 1822. Plants heterozygous or homozygous for T are indicated as mutants, while the homozygotes G/G correspond to the wild-type. Total linalool content in the whole FEM aromatic core collection classified according to the *VvDXS1* genotype at position 1822 **(C)** and in a subset of accessions with the muscat mutation at SNP1822 classified according to the genotype at the marker FAH1 on chromosome 10 (Battilana et al., 2009; Emanuelli et al., 2011) **(D)**.

The concentration of monoterpenes and sesquiterpenes in the grapevine collection is shown in Figures 5A,B. The mean content of monoterpenes (in their free and glycosidically bound fractions and as a total) proved to be significantly different (*p* = 0.000) between homozygotes T/T or heterozygotes G/T for SNP1822 and wild-type homozygotes G/G, as assessed by both ANOVA and Kruskal-Wallis analyses. In particular, out of 70 accessions homozygous or heterozygous for T at position 1822, 62 had a total monoterpene content higher than 4 mg/kg of berries, which is typical of intensely flavored muscats in the classification scheme by Mateo and Jiménez (2000), while only eight cultivars (e.g., Italia and Perlette) showed a monoterpene content ranging from 1 to 4 mg/kg, which is attributed to non-muscat but aromatic varieties by the same authors. In contrast, all 17 homozygotes 1822 G/G proved to be neutral with a monoterpene content lower than 1 mg/kg of berries (Figure 5A). A similar, though lower, effect of the *VvDXS1* genotype was observed on the sesquiterpene content (Figure 5B), which represents a novel finding.

#### Terpenoid content in mature berries of transformed and WT microvine plants

**Figure 6:**
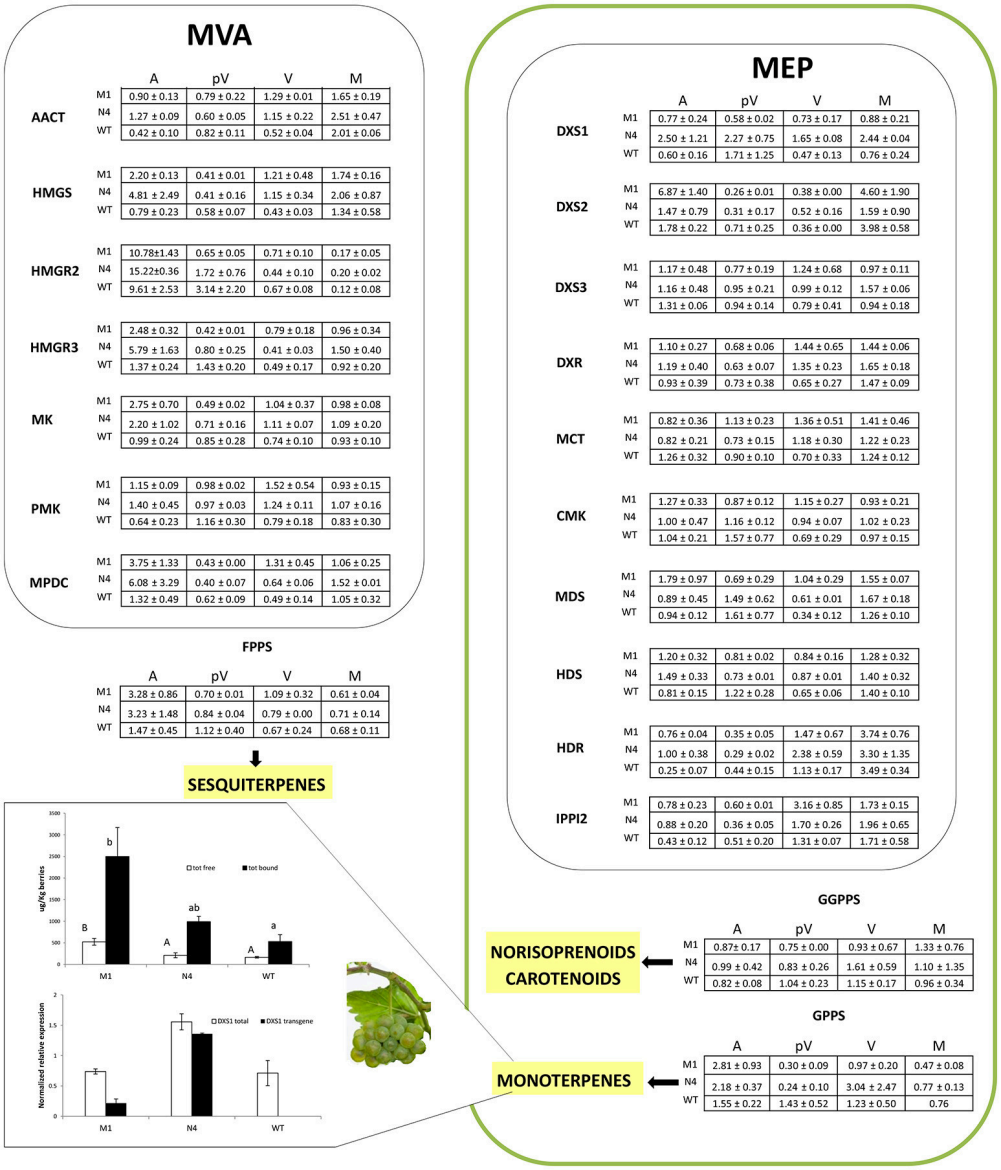
Transcription profile of specific genes of the MEP and MVA pathways during berry development (see matrix) and monoterpene content vs. *VvDXS1* transcript level in mature fruits (see histograms). For the time-course expression profiles, the raw Cq values from TaqMan card assay were elaborated according to Hellemans et al. (2007) and numbers in the cells are the mean of two biological replicates ± SE. The full gene name corresponding to each acronym can be retrieved in Figure 4. Monoterpene content was assessed in berry skin of fruits collected at technological maturity in 2016 (Table S2) and concentration values in the upper histogram are the mean ± SE of four biological replicates. The letters on the bars indicate different subsets according to ANOVA and Tukey's HSD *post-hoc* tests (*P* < 0.05). A portion of the skin powder grounded for metabolic analysis was used to quantify *VvDXS1* expression and values in the lower histogram are the mean ± SE of two (for WT) or three (for M1 and N4) biological replicates. M1 = Mi-M1, N4 = Mi-N4, A, flowers at anthesis; pV, berries at pre-*veraison*; V, berries at *veraison*; M, berries at maturity.

The monoterpene and sesquiterpene content was investigated in mature berry skins of transformed and control microvines cultivated in the greenhouse. Both free and bound monoterpenes were present at significantly higher levels in Mi-M1 compared to Mi-N4 and Mi-WT lines (Figure 6). Total bound monoterpenes (2500 μg/Kg for Mi-M1, 991 μg/Kg for Mi-N4, and 529 μg/Kg for Mi-WT) were more abundant than the total free component, which showed values near to 500 μg/Kg in Mi-M1 and far below in Mi-N4 and Mi-WT. The most abundant compounds were geranic acid (82% of both total free and bound monoterpenes in the Mi-M1 line), followed by citronellol, geraniol and nerol. Linalool was not present in any line (data not shown). Significant (*P* < 0.05) differences among lines were found for free geranic acid, bound geranic acid and citronellol, with the Mi-M1 line showing the highest level (Figure S6). No sesquiterpenes were detected in ripe berry skins of transformed and control microvines (data not shown). Samples were further evaluated for *VvDXS1* (transgene and endogenous gene) expression. A significantly decreased level was observed in Mi-M1 compared to Mi-N4 (six times less, Figure 6) confirming the significant differences seen between the two transformed lines in the leaf tissue (Figure 1C).

## Discussion

### The *VvDXS1* genotype is highly predictive of terpene content in grapevine

The first indications of the role that *VvDXS1* plays in the genetic control of monoterpene biosynthesis derived from the analysis of segregating progenies (Battilana et al., 2009; Duchêne et al., 2009) and were subsequently confirmed by a genetic association study based on berry taste (Emanuelli et al., 2010). In the present work a grapevine germplasm collection was considered, for which *VvDXS1* genotype and terpene content (as assessed by chemical methods) were analyzed in parallel. This analysis clearly highlighted that sequence variation of *VvDXS1* has a very high predictive value for the accumulation of monoterpenes and, as a novel finding, also of sesquiterpenes. The strength of such genetic effect is even more evident if considering that the investigated plants have been grown under field conditions and have been influenced by uncontrolled environmental factors.

### Transformed microvines carrying distinct *VvDXS1* alleles are reported for the first time

A metabolic engineering approach was adopted for studying the regulatory role of *VvDXS1* on terpenoid metabolism in grapevine, given its relevance for the quality of grapes and wines. Besides Chardonnay and Brachetto, which are frequently used cultivars amenable to genetic transformation (Iocco et al., 2001; Dalla Costa et al., 2009; Dhekney et al., 2011; Perrone et al., 2012), the microvine genotype was also employed. Several genetic experiments have used the microvine (Luchaire et al., 2017) since it was presented as a grapevine model system for functional genomic studies (Chaïb et al., 2010), however no studies based on engineered microvine plants have been published so far. The present work was focused on the microvine transformation with different *VvDXS1* alleles. The underlying goal was to investigate two potential perturbation mechanisms, the former based on a significant over-expression of the wild-type allele and the latter on the *ex-novo* expression of an enzyme with increased catalytic efficiency from the mutated allele. Here, we obtained a total of 13 transformed lines (nine microvines, two Chardonnay and two Brachetto) carrying the mutated or non-mutated form of *VvDXS1* gene with integrations ranging from a single copy to multiple copies (Figure 1, Figures S1, S2).

A high level of transgene expression was measured in the lines Mi-N4, C-M5, C-M6, and B-N1. Conversely, a low expression rate was observed in the other lines, likely due to multi-copy insertions that may produce silencing effects or to the “position effect,” which is related to the genomic region where the T-DNA copies are integrated. Both causes of epigenetic silencing are well documented in literature (Matzke and Matzke, 1998; De Buck et al., 2007; Tang et al., 2007) and nowadays they represent two of the major concerns in plant genetic engineering (Rajeevkumar et al., 2015).

The period required by 04C023V0006 microvine plants for flowering in the greenhouse (on average 197 days in Table 1) was four times longer than that reported by Chaïb et al. (2010) for two different microvine genotypes. Alternatively, the time needed to reach fruit maturity (berry sugar content >18 °Brix) was consistent with that observed by Chaïb (i.e., 16 weeks post anthesis). Comparing microvine with the conventional grapevine genotypes, we confirmed that the time necessary for flowering is dramatically reduced while fruit ripening times are very similar (data not shown). Moreover, the *VvDXS1* insertion did not affect the phenology or the morphology of the microvine plants (Table 1, Table S2).

### *VvDXS1* is spatially and developmentally regulated in the transformed microvine lines

The spatio-temporal analysis of the *VvDXS1* transcript in microvine confirmed that this gene is not expressed in a steady-state level in different grapevine organs and during fruit ripening but it is highly modulated as previously observed in Moscato Bianco (Battilana et al., 2011). Moreover, the *VvDXS1* expression trend in the transformed lines Mi-M1 and Mi-N4 was similar to that in the WT plant (Figure 6, Figure S4). The modulation of *VvDXS1* was even more evident when the expression profile of the endogenous gene and transgene were simultaneously analyzed in Mi-M1 and Mi-N4 lines (Figure 3, Figure S3). Under these conditions, the transgene proved to be controlled by the same organ-specific and developmental signals as the endogenous counterpart. In particular, a significantly higher expression was detected in the leaf with respect to the other tissues, an outcome which may be explained by the strong requirement of chlorophyll and carotenoids during leaf maturation (Estévez et al., 2000). Regarding the berry developmental stages, a significant increase was assessed after *veraison*, confirming the findings of Battilana et al. (2011). Such data may indicate additional levels of gene expression regulation occurring post-transcriptionally or with a feedback mechanism, as reported for other plant species (Hemmerlin et al., 2012).

### The integration of *VvDXS1* alleles in transformed microvines perturbs several terpenoid pathways at the transcriptional level

The TaqMan array, which was developed *ad hoc* to harbor as many as possible truly expressed genes, allowed to evaluate the transcription profile of a number of terpenoid pathway genes in the same technical conditions. Four important stages in the grape reproductive life were considered (Figure 4).

#### Flowering

It has been demonstrated that plants at anthesis emit a plethora of volatile terpenes to attract pollinating insects and for the protection of floral tissues from microbial pathogens or herbivores (Tholl, 2006). The overexpression of *Vv-N-DXS1* in Mi-N4 and, to a lesser extent, of *Vv-M-DXS1* in Mi-M1 at flowering time resulted in a significant up-regulation of *HDR* (VIT_03s0063g02030), which codes for the ultimate enzyme of the MEP pathway. Experimental evidence in several plants suggests that, in addition to *DXS*, this gene may have a rate-limiting role in IPP and DMAPP synthesis (Botella-Pavía et al., 2004; Page et al., 2004; Cordoba et al., 2009; Vranová et al., 2012) and in grapevine its expression has been reported to closely parallel the *veraison*-initiated accumulation of monoterpenes (Martin et al., 2012; Wen et al., 2015). On the contrary, *DXR* (VIT_17s0000g08390), another putative regulatory gene (Carretero-Paulet et al., 2006; Wungsintaweekul et al., 2008), showed no expression variation in transformed lines compared to WT. This conforms to the observation that the rate-limiting role of *DXR* varies among plant species and in different conditions (Cordoba et al., 2009). The expression of other MEP pathway genes were not significantly affected in transformed microvines, which is in agreement with the minimal effects of *DXS* overexpression on transcript levels of MEP pathway genes in Arabidopsis plants under natural conditions (Wright et al., 2014). Surprisingly, three important genes of the cytosolic MVA pathway, *AACT* (VIT_12s0057g01200), *HMGS* (VIT_02s0025g04580), and *HMGR3* (VIT_03s0038g04100), exhibited strong up-regulation in the transformed lines, especially in line Mi-N4, compared to the WT. This outcome was unexpected as the recently reported examples of exchange events between the two pathways concerned only precursor intermediates like IPP, GPP (geranyl diphosphate), and FPP (farnesyl diphosphate) (Hemmerlin et al., 2012; May et al., 2013; Pazouki and Niinemets, 2016). In addition, in the literature there is no supporting evidence for a transcriptional co-regulation of the two pathways (Wille et al., 2004; Vranová et al., 2013). At most, a cross-talk regulation, if any, is expected to occur at a post-transcriptional level (Hemmerlin et al., 2012). Our observation adds to recent reports that indicate that there is deviation from the general notion that synthesis of sesquiterpenes and triterpenes occur via the MVA pathway whereas monoterpenes and diterpenes are synthetized via the MEP pathway, and instead suggests that these pathways are integrated rather than mutually exclusive (Chaurasiya et al., 2012).

Analysis of isoprenoid pathways downstream of IPP and DMAPP revealed a slight but widespread up-regulation of carotenoid, monoterpene and sesquiterpene pathway genes in transformed lines compared to the control. Several genes involved in carotenoid metabolism were induced, although not significantly, in both Mi-N4 and Mi-M1 lines with respect to WT. Similarly, overexpression of *DXS* in other plant species resulted in an increased expression of genes responsible for carotenogenesis (e.g., phytoene synthase) and a higher carotenoid content (Estévez et al., 2001; Morris et al., 2006; Henriquez et al., 2016; Simpson et al., 2016). Moreover, a recent genome-wide association study investigating the variation for carotenoid concentration in maize grain identified *DXS1* as a candidate gene (Suwarno et al., 2015). It is also interesting to observe the significantly higher level of *CCD4* transcript (VIT_02s0087g00910) in Mi-N4 line compared to WT. CCD4 is a member of carotenoid cleavage dioxygenases (CCDs), which are involved in norisoprenoid production. Norisoprenoids are found in flowers and fruits of many plants and possess aromatic properties together with low odor thresholds (Schmidt et al., 2006; Ebeler and Thorngate, 2009). They also contribute to floral and fruity aroma in Muscat cultivars and in Riesling-type varieties (Baumes et al., 2002). Pertaining to monoterpenes, a strong up-regulation was observed for ocimene synthase (VIT_12s0134g00020) in both transformed lines but more prominently in Mi-N4. In grapevine, ocimene synthase is specifically expressed in flower buds and to a lesser extent in open flowers (Lücker et al., 2004; Matarese et al., 2014). Regarding sesquiterpenes, a significant up-regulation was noticed for valencene synthase (VIT_18s0001g04050), which generates one of the major volatiles emitted from flowers of white and red varieties (Lücker et al., 2004; Martin et al., 2009).

Our findings seem to indicate that the transcription of genes which are physiologically “turned on” during anthesis is strongly enhanced in transformed plants with a potentially stronger MEP pathway flux. However, while it is reasonable to connect the enhanced expression of carotenoid-, apo-carotenoid-, and monoterpene pathway genes to the increased MEP pathway flux, the cause of an enhanced expression of MVA and, as a consequence, of sesquiterpene genes remains unknown and shall be the subject of further investigation.

#### Pre-veraison

At pre-*veraison* a diffused down-regulation of MEP and MVA pathway genes was observed in transformed lines compared to WT. A similar pattern was seen for carotenoid genes while monoterpene and sesquiterpene synthase transcripts were poorly detected in all the analyzed plants.

#### Veraison and maturity

At *veraison*, most of the genes were up-regulated in the transformed lines respect to WT. According to Coombe and McCarthy (2000), at this stage several physiological and biochemical processes are initiated, and during the subsequent ripening phase major aromatic compounds including terpenes and norisoprenoids are synthesized. As discussed for the anthesis stage, MEP and MVA pathway genes showed a widespread rise in the transformed lines compared to the control. Regarding the carotenoid pathway, many genes were modulated: the first genes of the route showed a high expression in transformed lines while a more heterogeneous pattern was detected for the genes responsible for carotenoid degradation.

At maturity, MEP and MVA pathway genes were poorly modulated between the transformed lines and the control. Regarding sesquiterpenes, germacrene-D-synthase (VIT_18s0001g04280) was strongly expressed in Mi-M1 compared to WT.

### Overexpression of *VvDXS1* increases the level of monoterpenes in ripe berries with the form N284 being more effective

The microvine system has allowed to evaluate the metabolic profile of fruits engineered for an important regulatory gene of terpenoid metabolism. We detected significant differences between transformed and control grapes in the accumulation of monoterpenes at harvest time. In particular, the total monoterpene content was 1.7- and 4.4-fold in Mi-N4 and Mi-M1 lines with respect to WT microvines, with ratios ranging from 1.3 to 1.9 (Mi-N4) and from 3.2 to 4.7 (Mi-M1) for free and glycosidically bound monoterpenes, respectively (Figure 6). These values are similar, albeit often higher in the case of Mi-M1, to those reported in previous experiments assessing the enhancement of isoprenoid compounds upon *DXS* overexpression in other plants (Lois et al., 2000; Estévez et al., 2001; Enfissi et al., 2005; Carretero-Paulet et al., 2006; Morris et al., 2006; Muñoz-Bertomeu et al., 2006; Peebles et al., 2011; Vaccaro et al., 2014; Wright et al., 2014; Shi et al., 2016; Simpson et al., 2016; Zhou et al., 2016; Jadaun et al., 2017).

Even if we cannot exclude an effect of the *gai* mutation on terpene biosynthesis (Hong et al., 2012; Murcia et al., 2017), our findings conform with the idea that *DXS1* ectopic expression can raise the metabolic flux through the MEP pathway, thereby improving the formation of isoprenoids. Comparing Mi-N4 with WT plants, a close relationship may be observed between the increase in *DXS1* total transcripts (2.2-fold) and its end products (1.7-fold increase in total monoterpene content) (Figure 6). Oppositely, in the case of Mi-M1 line, a weak expression of the *DXS1* mutated allele resulted in an important gain in total monoterpenes (4.4-fold increase) (Figure 6). This outcome is in line with the results of Battilana et al. (2011) who found improved catalytic performances of the muscat DXS1 enzyme in comparison with the neutral form resulting in enhanced monoterpene biosynthesis.

The effect played by *VvDXS1* on sesquiterpene content in the FEM aromatic core collection could not be confirmed in the microvine lines analyzed here, as they did not accumulate sesquiterpenes in ripe berry skins. A possible explanation is that the microvines have been grown in a greenhouse (according to the extant restrictions on GMOs in Italy), whereas the germplasm collection is planted in open field. Although the investigated microvines proved to be a convenient model system to detect differences in terpenoid pathway genes and monoterpene content among lines, it is evident that such system maintained in the greenhouse does not reproduce exactly the environmental conditions present in the vineyard (especially the exposure to light). Moreover, additional genes might play a limiting role in terpene production in the microvine genetic background. Both facts might also explain the lower monoterpene content of the transformed microvines (Figure 6) compared to the monoterpene content of the germplasm accessions with the muscat mutation (Figure 5A). An effect of *VvDXS1* on sesquiterpene biosynthesis cannot be excluded in flowers, which represent the only tissue where a significant modulation of sesquiterpene pathway genes was observed (Figure 4). However, the quantification of sesquiterpenes in flowers was out of the aim of the present study.

### Prospects for the bioengineering of isoprenoid biosynthesis in grapevine

The metabolic engineering approach adopted in the present work provided new insights into the functional effect of *VvDXS1* alleles on terpenoid metabolism in grapevine. In order to optimize this basic approach from a biotechnological point of view, one should keep in mind some important aspects. First, *DXS1* is post-transcriptionally regulated both at the level of gene expression (e.g., the *DXS* down-regulation by PSY activity and carotenoid synthesis) and of protein abundance/activity (e.g., the feedback inhibition of *DXS1* activity by IPP and DMAPP), which is especially important *in vivo* to rapidly link the pathway with environmental and physiological challenges or metabolic fluctuations (Lois et al., 2000; Banerjee et al., 2013; Hemmerlin, 2013; Ghirardo et al., 2014; Pokhilko et al., 2015; Rodríguez-Concepción and Boronat, 2015). Secondly, the MEP pathway flux may be diverted via cross-talk with the MVA route (Hemmerlin et al., 2012; Pazouki and Niinemets, 2016) or via export of intermediates like methylerythritol cyclodiphoshate (MecPP), as observed in transformed Arabidopsis plants overexpressing *DXS* (Xiao et al., 2012; Wright et al., 2014; González-Cabanelas et al., 2015), and hydroxymethylbutenyl diphosphate (HMbPP) (Ward et al., 2011). Finally, other limiting steps in the MEP-pathway or in upstream and downstream pathways may exist, which includes the need for coordination between plant development and secondary metabolite production in order to not compete for carbon sources (Estévez et al., 2001; Enfissi et al., 2005; Muñoz-Bertomeu et al., 2006; Rodríguez-Concepción and Boronat, 2015; Shi et al., 2016; Zeng et al., 2016). In this regard, the analysis of additional metabolites (e.g., carotenoids and chlorophylls) could point out potentially competing reactions. The involvement of multiple key enzymes might also explain the lack of linalool in the microvines under investigation; in particular, terpene synthases or other genes in the confidence interval of the previously identified linalool-specific QTL on chromosome 10 (Battilana et al., 2009; Emanuelli et al., 2011; Costantini et al., 2017) might play a limiting role in linalool production in combination with *VvDXS1*, as highlighted in the grapevine germplasm (Figures 5C,D) and reported in other plants (Zeng et al., 2016).

## Conclusion

The genetic transformation of microvine plants with *VvDXS1* causes a significant perturbation in downstream pathways both at the transcriptional and metabolic level with no evident effect on plant morphology and phenology. The increased production of monoterpenes in the transformed lines with respect to the control may be predominantly attributable to the increased activity of the VvDXS1 enzyme with the K284N mutation and to a lesser extent due to *VvDXS1* up-regulation. This gene is therefore an effective target for improving metabolic flux in the monoterpene biosynthetic route and accumulating more aroma-active compounds in the grape berry. Moreover, our experiment has shown a potential effect of *VvDXS1* on the sesquiterpene pathway. The continuation of this study will enable the evaluation of the *VvDXS1* gain-of-function mutation on the level of additional metabolites in the microvine model system under control and stress conditions.

## Author contributions

FE and MSG designed the project; FE and SL developed the TaqMan array tool; LDC performed microvine transformation, and contributed with MT to management and characterization of plant materials at morphological and transcriptional level; PM-S and SL genotyped the FEM aromatic core collection and prepared the samples for metabolic analysis; SM, DS, and RL provided the metabolic data; EC and AC did the bioinformatic analysis; IG was responsible for Brachetto and Chardonnay engineering with a contribution by LC; LDC, FE, MT, PM-S, LC, MM, and MSG took part in data interpretation; LDC drafted the work; LC, FE, and MSG revised it critically. All the authors approved the final version of this text.

### Conflict of interest statement

The authors declare that the research was conducted in the absence of any commercial or financial relationships that could be construed as a potential conflict of interest.
