# Supplementary material for: Induction of Terpene Biosynthesis in Berries of Microvine Transformed with VvDXS1 Alleles
Source: Front Plant Sci. 2018 Jan 17;8:2244. doi: 10.3389/fpls.2017.02244 (PMC5776104; doi:10.3389/fpls.2017.02244)
Supplement: Supplementary file 1 [file DataSheet1.PDF]

Supplementary materials and methods

Gene expression analysis

The qPCR was carried out on a LightCycler® instrument (Roche Diagnostics, Basel, Switzerland) in 20 µl volume containing LC 480 SYBR Green I Master Mix (Roche Diagnostics, Basel, Switzerland), 0.5 µM primers (Table S4) and 5 µl of diluted cDNA (1:30). An initial denaturation step at 95 °C for 5 min was followed by 45 cycles at 95 °C for 15 s, 58 °C for 30 s and 72 °C for 10 s. Finally, to detect non-specific amplification in cDNA samples a melting curve analysis was performed as follows: 95 °C for 10 s, 55 °C for 15 s and a stepwise T increase (0.06 °C/s) up to 95 °C with a continuous detection. The experiments were run in duplicate for each data point. Primer efficiency was obtained by means of a standard curve built using a 1:5 serial dilution of cDNA for a total of 4 points. The efficiency (E) was calculated with the equation $E=10^{(-1/slope)}$. Gene expression data were analyzed according to the relative quantification model published by Hellemans *et al.* (2007). The mean value of wild-type control or the mean value of all the samples were alternatively used as the quantification cycle (Cq) reference value in the calculation of the delta-Cq. Two housekeeping genes, glyceraldehyde 3-phospate dehydrogenase (*GAPDH*) (Reid *et al.*, 2006) and actin (*ACT*) (Gatto *et al.*, 2008) were used for determining a normalization factor.

Normalized cDNA libraries

Sampling

Berries from four grapevines (*Vitis vinifera* cv Gewürztraminer (TRA), Malvasia di Candia aromatica (MAL), Moscato Bianco (MOB) and Rhein Riesling (RIE)) were collected during the 2012 growing season. MOB, RIE and TRA were sampled at four developmental points: berry softening (E-L 35), intermediate brix (E-L 36), quite ripe (E-L 37), harvest ripe (E-L 38), based on the modified E-L system for grapevine growth stages defined by Coombe (1995). MAL was sampled only at three stages (E-L 35,-E-L 36 and E-L 37) because berries failed to ripen over 18° Brix (harvest ripe). At each time point, ten bunches were taken from ten plants out of the ~ 250 grown in the experimental field “Inferno” of FEM (Fondazione Edmund Mach, San Michele all’Adige, Italy). Care was paid to sample from different vines and positions within each vine. In the laboratory, berries were pooled in order to minimize environmental effects and then divided into two batches. Berries from the first batch were hand-peeled, the skins were immediately frozen in liquid nitrogen and stored at -80 °C until RNA extraction. Berries from the second batch were homogenized to juice (80 mL) and analyzed for titratable acidity and soluble solids content by FT-

IR (Fourier Transform InfraRed) spectroscopy with a FOSS instrument (FOSS NIRSystems, Oatley, Australia).

RNA extraction

Total RNA was extracted from grape skins using the Spectrum™ Plant Total RNA Kit (Sigma-Aldrich, St. Louis, Missouri, USA). RNA quantity and quality were evaluated with a NanoDrop ND-8000 spectrophotometer (NanoDrop Technologies, Wilmington, Delaware, USA) and an Agilent 2100 Bioanalyzer (Agilent Technologies, Mississauga, Ontario, Canada). The RNA extracted at the four (three for MAL) sampling points was then pooled for each cultivar.

Normalized random primed cDNA library for 454 sequencing

Ten micrograms of RNA of each pool were sent to Vertis Biotechnology AG (http://www.vertis-biotech.com/) for normalized library construction. From the DNase treated RNA samples poly(A)+ RNA was isolated, which was used for cDNA synthesis. First-strand cDNA synthesis was primed with a N6 randomized primer. Then 454 adapters A and B were ligated to the 5' and 3' ends of the cDNAs. The cDNAs were finally amplified with PCR (12 cycles for MOB, RIE, TRA and 13 cycles for MAL libraries) using a proofreading enzyme. Libraries were normalized by hydroxylapatite chromatography, and the single-stranded cDNA was amplified by PCR (nine cycles). cDNA was then selected with gel fractioning for fragments of size from 600 to 800 bp.

454 sequencing

Sequencing was carried out in a Genome Sequencer GS FLX Titanium (Roche-454 Life Sciences, Brandford, CT, USA) available at FEM Genomics Platform, according to the manufacturer’s instructions. Each library (MAL, MOB, RIE and TRA) occupied one half of a picotiter plate. Raw reads were subsequently subjected to trimming, removal of adapters/primers and low quality reads using SeqTrim (minimum read length = 50 bp; number of errors allowed in MID = 2; average limit filter quality = 13; size window filter quality = 7; max undetermined nucleotides = 2).

Bioinformatic analysis

Data generated from 454 Life Sciences (Roche) were pre-processed to select reads for length and quality parameters before read alignment on the 12x V1 version of the *Vitis vinifera* genome (http://genomes.cribi.unipd.it/grape/) by SMALT tool (Fonseca *et al.*, 2012; Hatem *et al.*, 2013). A post-alignment step led to gene identification and coverage depth determination by SAMtools (Li *et al.*, 2009).

The minimum number of reads required to consider a gene as expressed was identified by negative binomial and chi-square tests. Once expressed genes were identified, they were functionally and structurally annotated with InterProScan (Quevillon *et al.*, 2005).

Candidate genes involved in terpenoid biosynthesis

A priori selection of candidate genes involved in the biosynthetic pathways of terpenoids in grapevine was performed by searching against the KEGG (Kyoto Encyclopedia of Genes and Genomes, http://www.genome.jp/kegg-bin/show_organism?org=vvi) database, which links genomic information with higher order functional descriptors. The selected genes were then checked for their expression in the normalized cDNA libraries; the read alignments were manually inspected to define a final set of sequences (genes and eventually splicing isoforms) to be further investigated by specific TaqMan probes (Applied Biosystems, Foster City, CA, USA).

Statistical analysis

All the statistical analyses reported in this work were performed with STATISTICA software version 9 (StatSoft, OK, USA).

References not included in the main text

Fonseca NA, Rung J, Brazma A, Marion, JC. 2012. Tools for mapping high-throughput sequencing data. Bioinformatics **28**, 3169–3177.

Gatto P, Vrhovsek U, Muth J, *et al.* 2008. Ripening and genotype control stilbene accumulationin healthy grapes. Journal of Agricultural and Food Chemistry **56**, 11773–11785.

Hatem A, Bozdağ D, Toland AE, Çatalyürek ÜV. 2013. Benchmarking short sequence mapping tools. BMC Bioinformatics **14**, 184.

Li H, Handsaker B, Wysoker A, *et al*. 2009. The Sequence Alignment/Map format and SAMtools. Bioinformatics **25**, 2078–2079.

Quevillon E, Silventoinen V, Pillai S, Harte N, Mulder N, Apweiler R, Lopez R. 2005. Nucleic Acids Research **33**, 116–120.

Reid KE, Olsson N, Schlosser J, Peng F, Lund ST. 2006. An optimized grapevine RNA isolation procedure and statistical determination of reference genes for real-time RT-PCR during berry development. BMC Plant Biology **6**, 27.
