# Supplementary material for: Induction of Terpene Biosynthesis in Berries of Microvine Transformed with VvDXS1 Alleles
Source: Front Plant Sci. 2018 Jan 17;8:2244. doi: 10.3389/fpls.2017.02244 (PMC5776104; doi:10.3389/fpls.2017.02244)
Supplement: Supplementary file 2 [file DataSheet2.PDF]

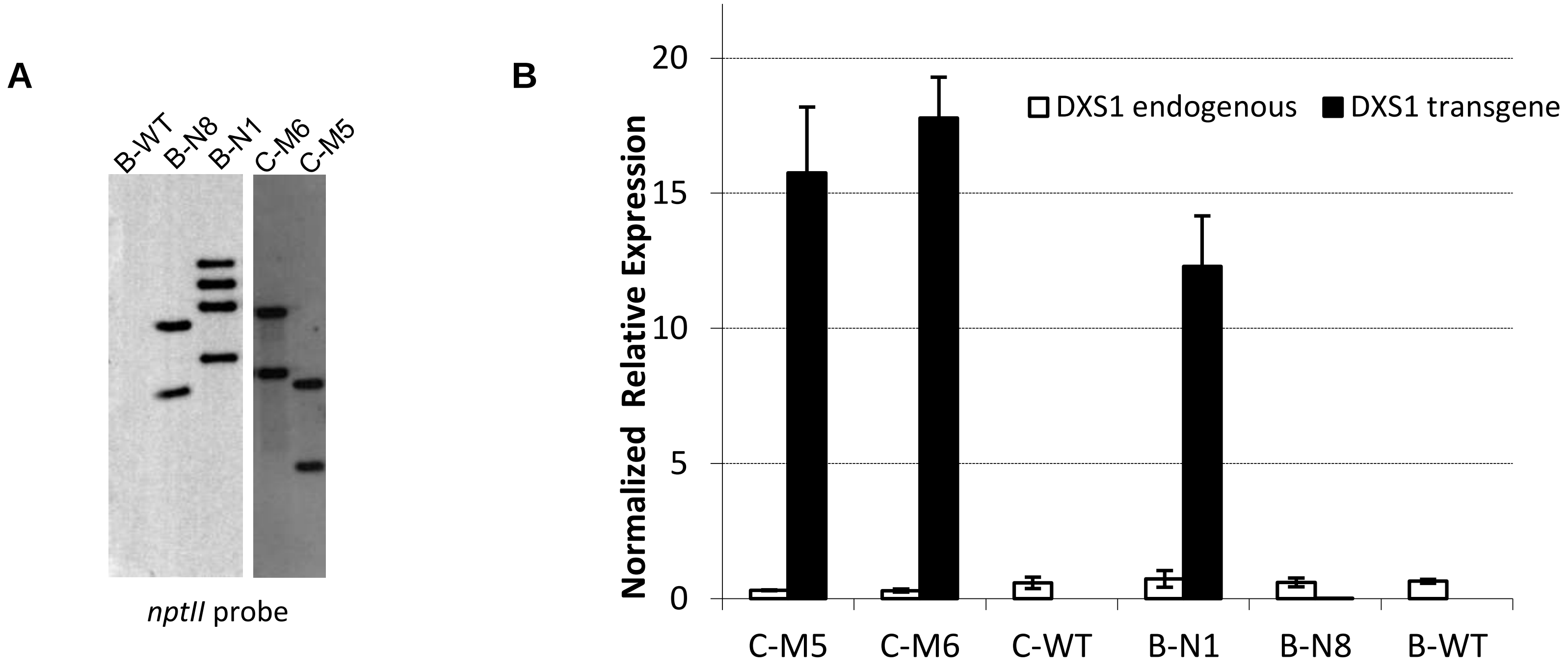

Figure S1. Molecular characterization of Brachetto (B) and Chardonnay (C) transformed plants in the greenhouse. (A) Determination of T-DNA integration copies by Southern blot with *nptII* probe. (B) Transcription profile of endogenous (white bars) and transgenic (black bars) *VvDXS1* in the leaf tissue. Expression values are the mean ± SE of two biological replicates (three in the case of C-M5 and C-M6). Abbreviations: WT = wild-type, N = neutral and M = muscat *VvDXS1* allele.
