# Supplementary material for: Induction of Terpene Biosynthesis in Berries of Microvine Transformed with VvDXS1 Alleles
Source: Front Plant Sci. 2018 Jan 17;8:2244. doi: 10.3389/fpls.2017.02244 (PMC5776104; doi:10.3389/fpls.2017.02244)
Supplement: Supplementary file 3 [file DataSheet3.PDF]

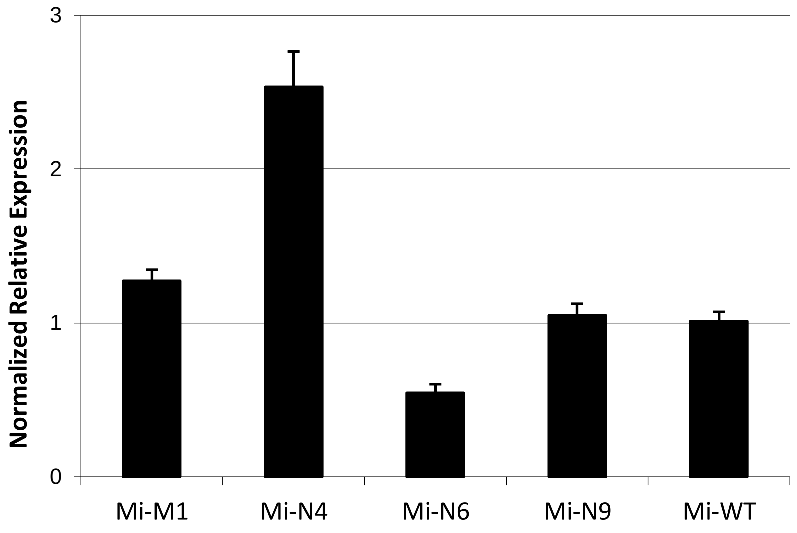

Figure S2. Expression analysis of total (endogenous and transgenic) *VvDXS1* in various *in vitro* microvine lines. Expression values are the mean ± SE of three biological replicates (two in the case of Mi-N4) analyzed in two separate PCR sessions.
