# Supplementary material for: Induction of Terpene Biosynthesis in Berries of Microvine Transformed with VvDXS1 Alleles
Source: Front Plant Sci. 2018 Jan 17;8:2244. doi: 10.3389/fpls.2017.02244 (PMC5776104; doi:10.3389/fpls.2017.02244)
Supplement: Supplementary file 4 [file DataSheet4.PDF]

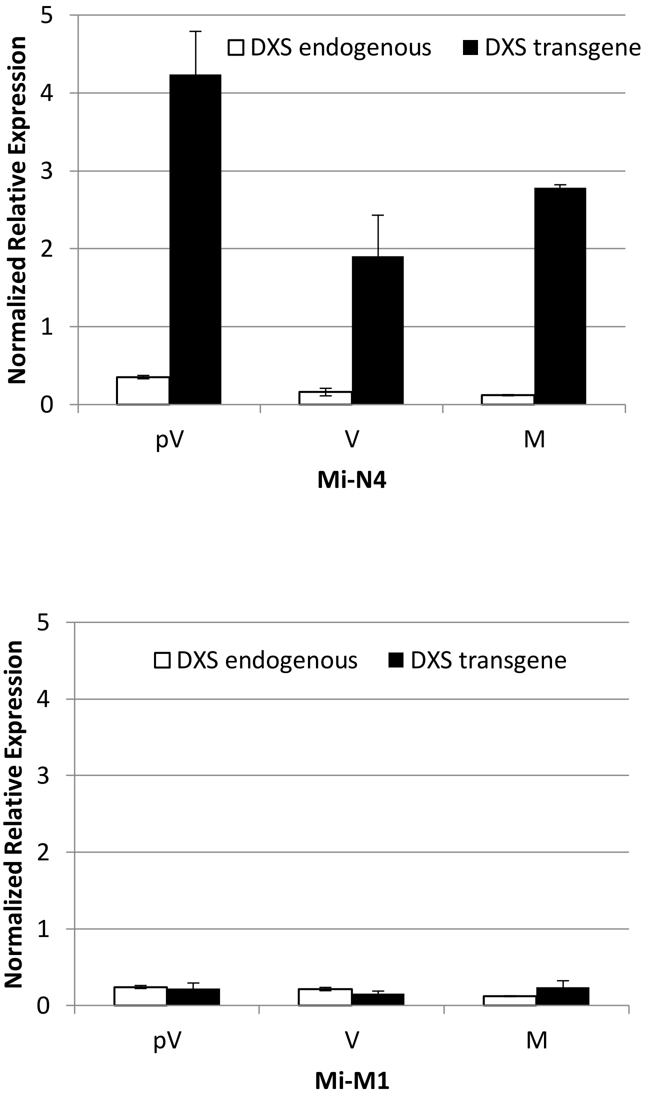

Figure S3. Expression analysis of endogenous (white) and transgenic (black) *VvDXS1* in berries sampled at different developmental stages from the Mi-N4 and Mi-M1 plants. The same cDNAs employed for the TaqMan card assay were assessed here. Expression values are the mean ± SE of two biological replicates. Abbreviations: pV = berries at pre-*veraison* stage, V = berries at *veraison* stage, M = berries at technological maturity (18 °Brix).
