# Supplementary material for: Induction of Terpene Biosynthesis in Berries of Microvine Transformed with VvDXS1 Alleles
Source: Front Plant Sci. 2018 Jan 17;8:2244. doi: 10.3389/fpls.2017.02244 (PMC5776104; doi:10.3389/fpls.2017.02244)
Supplement: Supplementary file 5 [file DataSheet5.PDF]

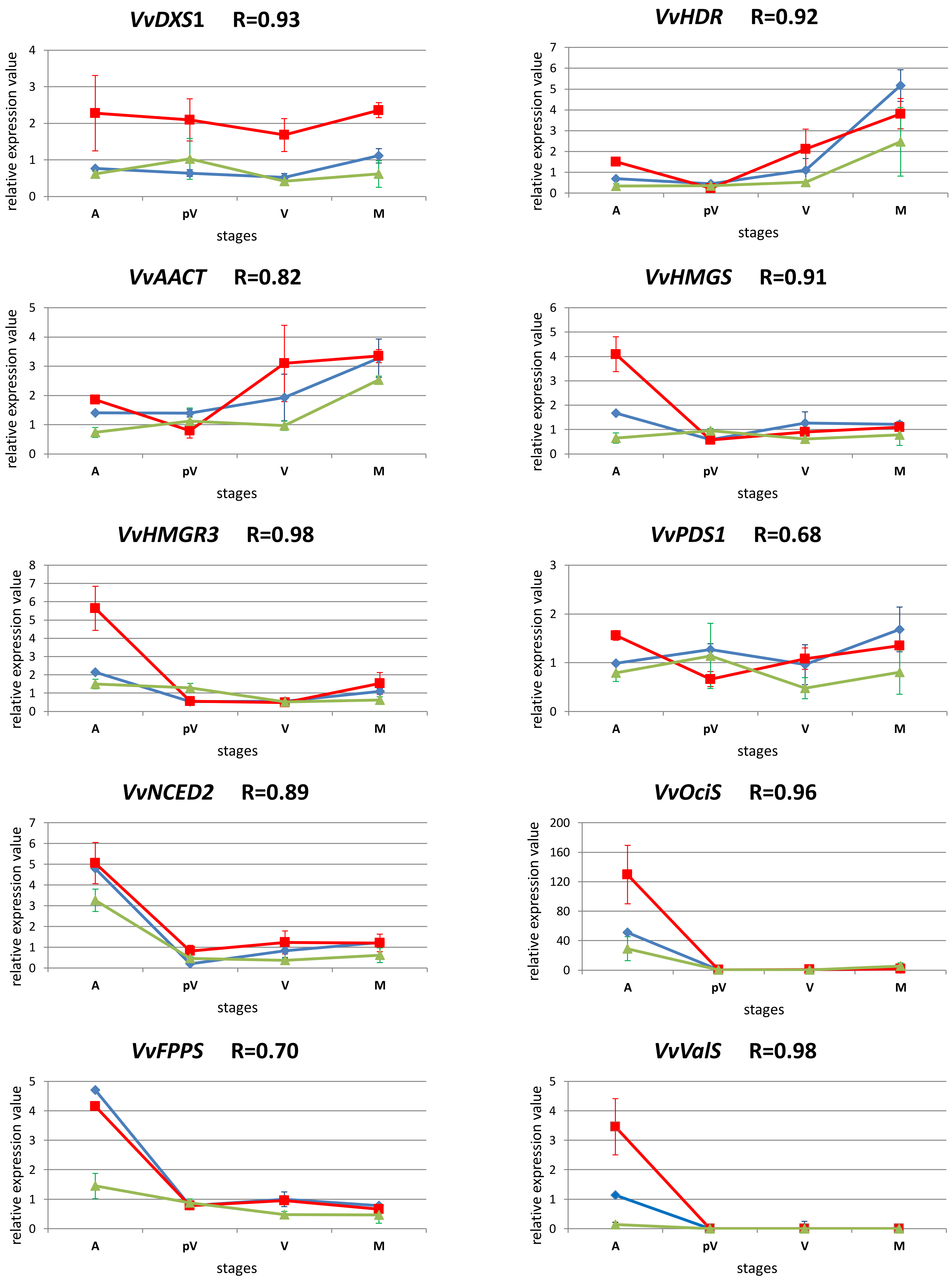

Figure S4. Transcriptional profiling during berry development of a set of terpenoid pathway genes in transformed and WT microvines. The same cDNAs employed for the TaqMan card assay were assessed through qPCR by using primers designed *ad-hoc* for the amplification with sybr-green (Table S4). The correlation between the two sets of measurements is indicated by Pearson correlation coefficient (R). qPCR expression values are the mean ± SE of two biological replicates both analyzed in duplicate. Data are normalized to the reference genes actin and glyceraldehyde-3-phosphate dehydrogenase. Blue = Mi-M1 line, red = Mi-N4 line, green = Mi-WT line. Abbreviations: A = flowers at anthesis, pV = berries at pre-*veraison*, V = berries at *veraison*, M = berries at maturity.
