# Supplementary material for: Induction of Terpene Biosynthesis in Berries of Microvine Transformed with VvDXS1 Alleles
Source: Front Plant Sci. 2018 Jan 17;8:2244. doi: 10.3389/fpls.2017.02244 (PMC5776104; doi:10.3389/fpls.2017.02244)
Supplement: Supplementary file 6 [file DataSheet6.PDF]

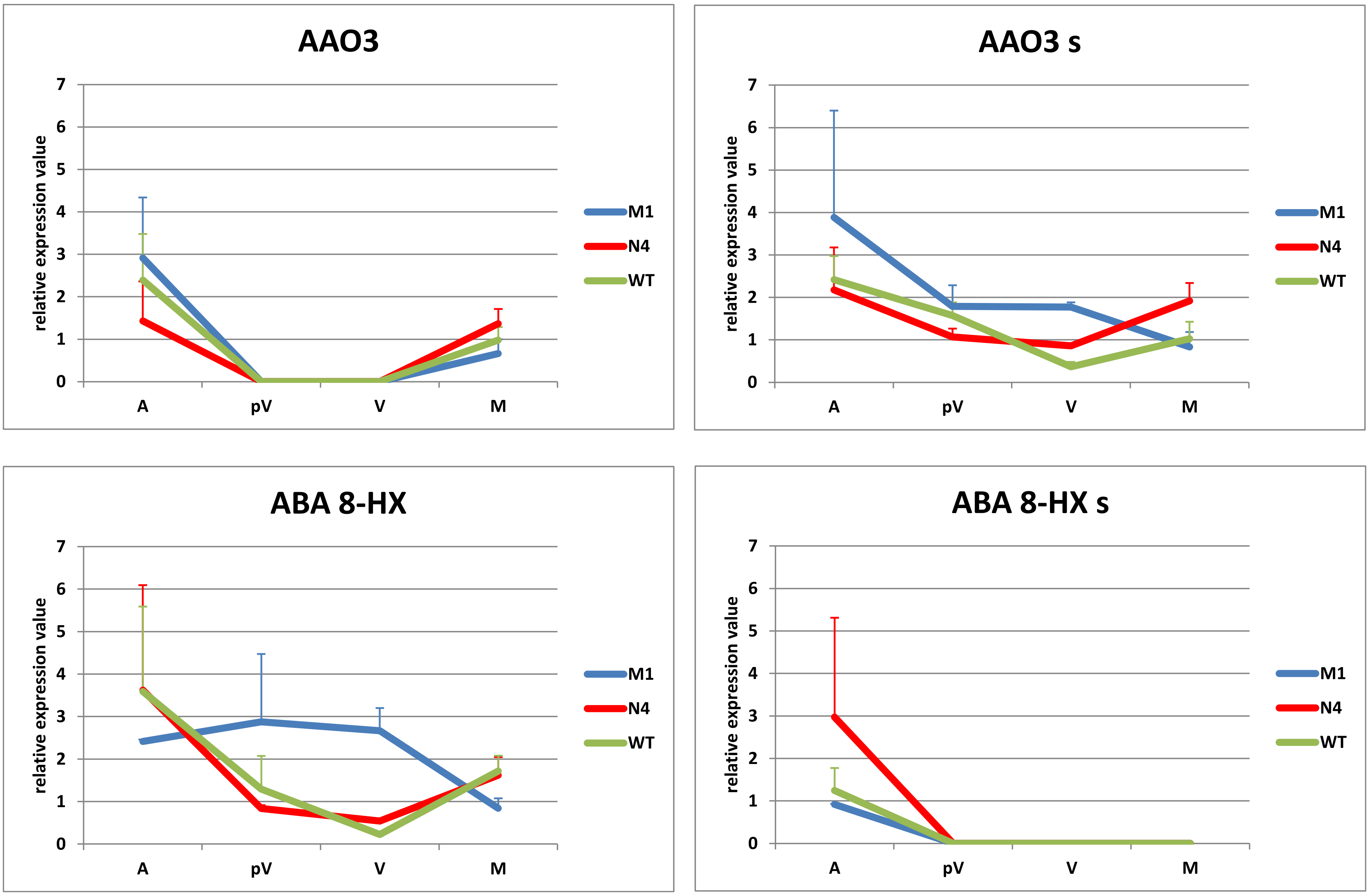

Figure S5. Transcriptional profiling of two genes with alternative splicing as assayed through TaqMan array cards in transformed and WT microvines during berry development. Expression values are the mean ± SE of two biological replicates both analyzed in duplicate. Data are normalized to the reference genes actin and glyceraldehyde-3-phosphate dehydrogenase. Abbreviations: s = splicing variant, M1 = Mi-M1, N4 = Mi-N4, WT = Mi-WT, A = flowers at anthesis, pV = berries at pre-*veraison*, V = berries at *veraison*, M = berries at maturity.
