# Supplementary material for: Induction of Terpene Biosynthesis in Berries of Microvine Transformed with VvDXS1 Alleles
Source: Front Plant Sci. 2018 Jan 17;8:2244. doi: 10.3389/fpls.2017.02244 (PMC5776104; doi:10.3389/fpls.2017.02244)
Supplement: Supplementary file 7 [file DataSheet7.PDF]

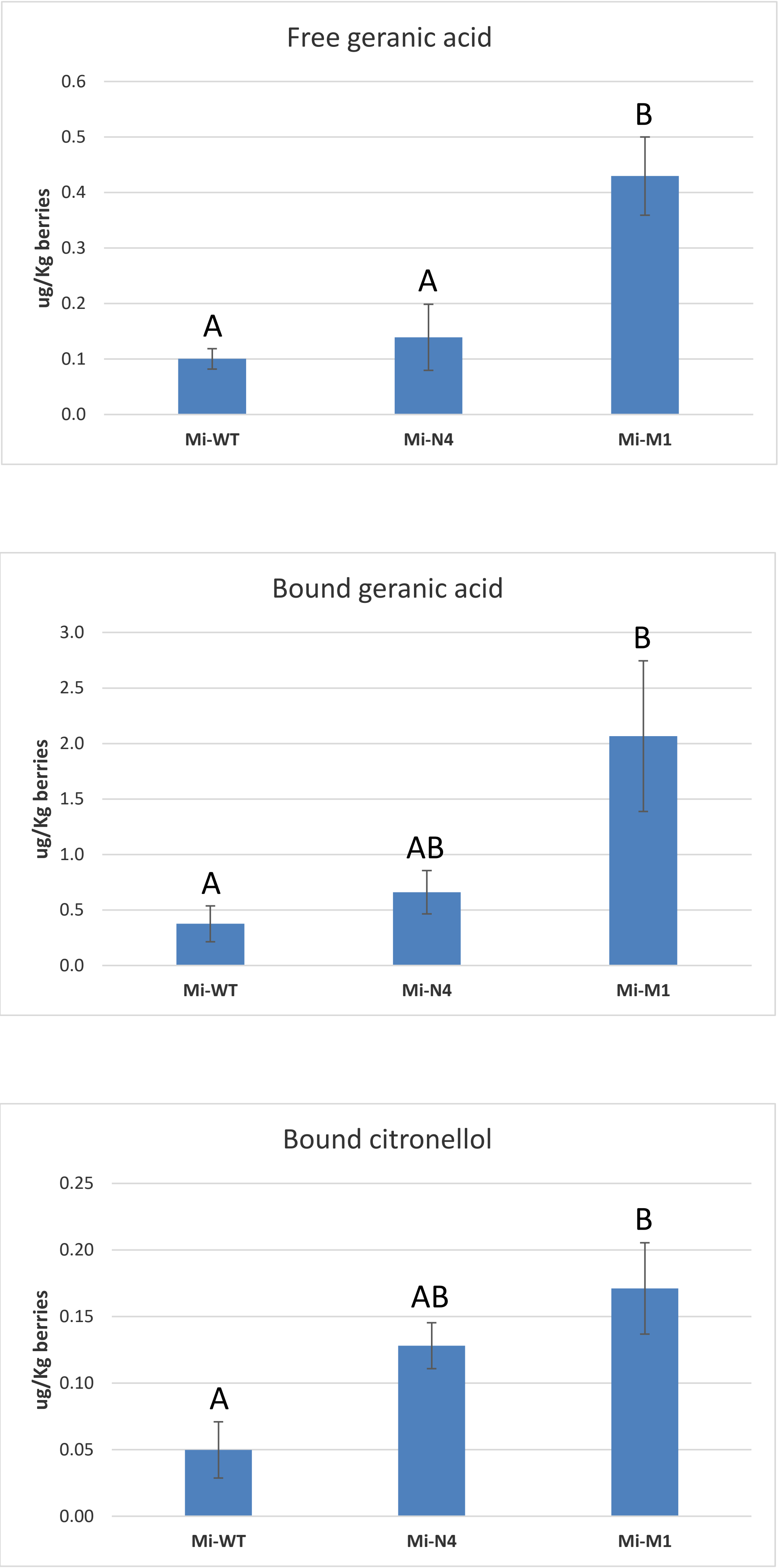

Figure S6. Accumulated monoterpenes in berry skin showing a significantly different level among microvine lines. Fruits were collected at technological maturity in 2016 (Table S2). Concentration values are the mean ± SE of four biological replicates. The letters on the bars indicate different subsets according to ANOVA and Dunn's post-hoc test ($P<0.05$).
