# Supplementary material for: Induction of Terpene Biosynthesis in Berries of Microvine Transformed with VvDXS1 Alleles
Source: Front Plant Sci. 2018 Jan 17;8:2244. doi: 10.3389/fpls.2017.02244 (PMC5776104; doi:10.3389/fpls.2017.02244)
Supplement: Supplementary file 8 [file DataSheet8.PDF]

Table S1. List of the genes assayed through the TaqMan array cards. Gene ID and description correspond to the 12x V1 version of the *Vitis vinifera* genome (http://genomes.cribi.unipd.it/grape/). The most widely adopted acronym is reported within brackets for each gene. Splicing variants are marked with an asterisk; endogenous genes are italicized. Each single assay (probe and primers) can be ordered to the manufacturer by using the "TaqMan ID".

**TaqMan ID**	**Gene description**	**Gene ID**
AIGJQKQ	1-deoxy-D-xylulose-5-phosphate synthase 2 (DXS2)	VIT_00s0218g00110
AIS08AZ	(3S)-linalool/(E)-nerolidol synthase VvTPS56 (VvPNLinNer2)	VIT_00s0271g00060
AIVI4NF	(3S)-linalool/(E)-nerolidol /(E,E)-geranyl linalool synthase VvTPS58 (VvPNLNGI3)	VIT_00s0372g00070
AIRR94R	(3S,6E)-nerolidol synthase 1 VvTPS54 (VvPNLinNer1)	VIT_00s0385g00010
AIWR2TN	(3S,6E)-nerolidol synthase 1VvTPS54 (VvPNLinNer1) * splicing variant	VIT_00s0385g00010
AIKAK3F	Acetyl-CoA acetyltransferase, cytosolic 2 (AACT)	VIT_00s0531g00050
AIT96G7	(3S)-linalool/(E)-nerolidol /(E,E)-geranyl linalool synthase VvTPS61 (VvPNLNGI4)	VIT_00s0572g00010
AI6RN7F	Phosphomevalonate kinase (PMK)	VIT_02s0012g02530
AI6RN7D	β-carotene hydroxylase 2 (CHY-B)	VIT_02s0025g00240
AI20TOQ	2-C-methyl-D-erythritol 2,4-cyclodiphosphate synthase (MDS)	VIT_02s0025g00370
AI1RVII	2-C-methyl-D-erythritol 2,4-cyclodiphosphate synthase (MDS)	VIT_02s0025g00380
AIKAK3G	Hydroxymethylglutaryl-CoA synthase (HMGS)	VIT_02s0025g04580
AIVI4NG	ABA 8'-hydroxylase (CYP707A1)	VIT_02s0087g00710
AIAAZPM	9-cis-epoxycarotenoid carotenoid cleavage dioxygenase 4 (CCD4)	VIT_02s0087g00910
AIPADSA	Squalene synthase (SS)	VIT_03s0038g00450
AIFASEK	Geranylgeranyl pyrophosphate synthase (GGPPS)	VIT_03s0038g03050
AIWR2TM	3-hydroxy-3-methylglutaryl coenzyme A reductase 3 (HMGR3)	VIT_03s0038g04100
AI5IP06	4-hydroxy-3-methylbut-2-enyl diphosphate reductase (HDR)	VIT_03s0063g02030
AIN1FL2	Squalene monooxygenase (SMO)	VIT_03s0088g01150
AII1MW8	CYP706A12 – MAX1- cytochrome P450	VIT_04s0008g01100
AILJI9O	YebC	VIT_04s0008g03370
AII1MW6	1-deoxy-D-xylulose-5-phosphate synthase 3 (DXS3)	VIT_04s0008g04970

AIX00ZU	Isopentenyl-diphosphate δ-isomerase 2 (IPPI2)	VIT_04s0023g00600
AILJI9N	Violaxanthin de-epoxidase (VDE2)	VIT_04s0043g01010
AI1RVIK	*Actin 1 (ACT1)*	*VIT_04s0044g00580*
AIVI4NE	3-hydroxy-3-methylglutaryl-coenzyme A reductase 2 (HMGR2)	VIT_04s0044g01740
AIHSOQZ	Phytoene synthase (PSY)	VIT_04s0079g00680
AII1MW7	Phytoene synthase (PSY)<br>* splicing variant	VIT_04s0079g00680
AIGJQKS	Geranylgeranyl pyrophosphate synthase (GGPPS)	VIT_05s0020g01240
AIT96G6	BTB/POZ domain-containing protein (B/P DCP)	VIT_05s0020g01250
AIMSHFU	1-deoxy-D-xylulose-5-phosphate synthase 1 (DXS1)	VIT_05s0020g02130
AICSV13	9-cis-epoxycarotenoid dioxygenase 6 (NCED6)	VIT_05s0051g00670
AIMSHFW	15-cis-ζ-carotene isomerase (CRTISO-Z)	VIT_05s0062g01110
AIPADSB	Farnesyltransferase/geranylgeranyltransferase type-1 subunit α (PLP)	VIT_05s0102g00230
AI39RU0	*SAND*	*VIT_06s0004g02820*
AI39RUY	4-hydroxy-3-methylbut-2-en-1-yl diphosphate synthase (HDS)	VIT_06s0004g02900
AI20TOS	*Elongation factor 1-α (EF1-α)*	*VIT_06s0004g03240*
AIRR94S	Abscisic acid 8'-hydroxylase 2 (ABA-HX)	VIT_06s0004g05050
AI39RUX	Abscisic -aldehyde oxidase 1 (AAO3)	VIT_06s0009g00770
AIT96G8	Abscisic -aldehyde oxidase 1 (AAO3)<br>* splicing variant	VIT_06s0009g00770
AI0IXCA	4-diphosphocytidyl-2-C-methyl-D-erythritol kinase (CMK)	VIT_06s0009g02320
AI1RVIH	Zeaxanthin epoxidase (ZEP) (ABA1)	VIT_07s0031g00620
AIY9Y54	Violaxanthin de-epoxidase (VDE1)	VIT_07s0031g01770
AIWR2TO	Carotene ε-monooxygenase (LUT1)	VIT_08s0007g04530
AI89KJU	Lycopene β cyclase (LCY-B)	VIT_08s0007g05690
AI5IP07	(3R)-linalool synthase VvTPS31 (VvPNRLin HM807390)	VIT_08s0007g06860
AICSV12	Prolycopene isomerase (CRTISO)	VIT_08s0032g00800
AID1T8A	Prolycopene isomerase (CRTISO)<br>* splicing variant	VIT_08s0032g00800
AIFASEJ	Phytoene desaturase 1 (PDS1)	VIT_09s0002g00100

AIX00ZW	Phytoene desaturase (PDS1)<br>* splicing variant	VIT_09s0002g00100
AIN1FL3	9-cis-epoxycarotenoid dioxygenase 2 (NCED2)	VIT_10s0003g03750
AI70MDM	Lycopene ε cyclase (LCY-E)	VIT_11s0016g01880
AIHSOQY	1-deoxy-D-xylulose-5-phosphate synthase 2 (DXS2)	VIT_11s0052g01240
AIBJXVW	Isopentenyl diphosphate δ-isomerase 2 (IPPI2)	VIT_11s0206g00020
AID1T8B	Phytoene desaturase 1 (PDS1)	VIT_12s0028g00710
AIGJQKR	Phytoene synthase (PSY)	VIT_12s0028g00960
AI89KJV	Prolycopene isomerase (CRTISO)	VIT_12s0035g01080
AI70MDN	Regulator of nonsense transcripts 3 (UPF3)	VIT_12s0035g01090
AIY9Y52	2-C-methyl-D-erythritol 4-phosphate cytidylyltransferase (MCT)	VIT_12s0035g01950
AIPADSC	Acetyl-CoA acetyltransferase, cytosolic (AACT)	VIT_12s0057g01200
AIQJBYK	Acetyl-CoA acetyltransferase, cytosolic (AACT)<br>* splicing variant	VIT_12s0057g01200
AI39RUZ	(E)-beta-ocimene synthase VvTPS34 (OciS)	VIT_12s0134g00020
AI5IP1A	Geraniol synthase VvTPS52 (VvPNGer)	VIT_12s0134g00140
AI0IXB9	Xanthoxin dehydrogenase ABA2 (ABA deficient 2)	VIT_13s0019g01010
AI70MDL	9,10[9',10']carotenoid cleavage dioxygenase 1 (CCD1)	VIT_13s0064g00840
AI89KJT	9,10[9',10']carotenoid cleavage dioxygenase 1 (CCD1)<br>* splicing variant	VIT_13s0064g00840
AI20TOR	Pinene synthase VvTPS44	VIT_13s0067g03790
AIQJBYJ	(-)-α-terpineol synthase (TerS)	VIT_13s0084g00010
AIAAZPN	Mevalonate diphosphate decarboxylase (MPDC)	VIT_13s0106g00790
AIMSHFV	ζ-carotene desaturase 1 (ZDS1)	VIT_14s0030g01740
AI6RN7E	Mevalonate kinase (MK)	VIT_14s0128g00330
AI5IP05	β-carotene hydroxylase 2 (CHY-B)	VIT_16s0050g01090
AIBJXVU	Geranylgeranyl diphosphate reductase (GGPPR)	VIT_17s0000g06280
AIX00ZT	1-deoxy-D-xylulose-5-phosphate reductoisomerase (DXR)	VIT_17s0000g08390
AILJI9M	1-deoxy-D-xylulose-5-phosphate reductoisomerase (DXR)<br>* splicing variant	VIT_17s0000g08390
AI5IP08	*Glyceraldehyde-3-phosphate dehydrogenase (GAPDH)*	*VIT_17s0000g10430*

AIY9Y53	Valencene synthase-like VvTPS01 (ValS)	VIT_18s0001g04050
AI1RVIJ	(-)-germacrene D synthase VvTPS07 (GerS)	VIT_18s0001g04280
AI0IXCB	Bergamotene synthase VvTPS10 (BerS)	VIT_18s0001g04780
AI20TOP	Abscisic acid 8'-hydroxylase (ABA 8’-HX)	VIT_18s0001g10500
AIS08A0	Abscisic acid 8'-hydroxylase 4 (ABA 8’-HX)<br>* splicing variant	VIT_18s0001g10500
AIQJBYI	Farnesyl diphosphate synthase (FPPS)	VIT_19s0015g01010
AID1T8C	Geranyl diphosphate synthase small subunit (GPPS ss)	VIT_19s0090g00530
AIHSOQ0	Geranyl diphosphate synthase small subunit (GPPS ss)<br>* splicing variant	VIT_19s0090g00530
AIBJXVV	9-cis-epoxycarotenoid dioxygenase 3 (NCED3)	VIT_19s0093g00550
AIRR94Q	Gibberellin 2-β-dioxygenase 1 (GA20X1)	VIT_19s0140g00120
AIS08AY	Gibberellin 2-β-dioxygenase 7 (GA20X7)	VIT_19s0177g00030
AI6RN7G	*Polyubiquitin-A (UBQ-A)*	*VIT_19s0177g00070*
