# Supplementary material for: Induction of Terpene Biosynthesis in Berries of Microvine Transformed with VvDXS1 Alleles
Source: Front Plant Sci. 2018 Jan 17;8:2244. doi: 10.3389/fpls.2017.02244 (PMC5776104; doi:10.3389/fpls.2017.02244)
Supplement: Supplementary file 9 [file DataSheet9.PDF]

Table S2. Main features of the four biological replicates of each microvine line (WT, N4 and M1) analyzed for monoterpene content at technological maturity, actions carried out to homogenize the ratio between total leaf area and berry number among plants and details about berry sampling. Abbreviations: M1 = Mi-M1, N4 = Mi-N4; [a] = measured on 20 April 2016; [b] = mean ± standard deviation (for at least three measures) of the leaves in opposite position respect to the clusters, measured on 29 April 2016; [c] = carried out on 4 May 2016; Fv/Fm = quantum yield of PSII photochemistry in the dark-adapted state; °Bx = degree brix of the pooled clusters (weighted mean); n.e. = not evaluated.

ID	Year of acclimation	Berry number[a]	Berry diameter[a] (mm)	Total leaf area[a] (cm^2)	(Fv/Fm)[b]	Operation for plant normalization[c]	Sampling date in 2016	Total n. of collected clusters	°Bx
WT1	2014	271	from 4 to 10 mm	3372	0.801 ± 0.02	cluster thinning	18, 24, 26 May	6	18.5
WT2	2014	190	from 4 to 10 mm	2969	0.803 ± 0.01	cluster thinning	17 May - 7, 8 June	4	18.3
WT3	2015	n.e.	n.e.	n.e.	n.e.	n.e.	24 May	1	18.5
WT4	2015	28	from 8 to 10 mm	2233	0.755 ± 0.01	leaf pruning	12, 18 May	2	18.6
N4-1	2015	80	from 6 to 12 mm	2362	0.796 ± 0.00	none	23 May	4	19.4
N4-2	2015	68	from 6 to 10 mm	1817	0.719 ± 0.06	none	23 May	5	19.7
N4-3	2014	30	from 5 to 10 mm	3261	0.790 ± 0.00	leaf pruning	23 May, 10 June	2	18.4
N4-4	2015	46	from 6 to 10 mm	1387	0.777 ± 0.02	none	26 May	2	18.9
M1-1	2015	117	from 5 to 10 mm	1352	0.783 ± 0.02	cluster thinning	12, 18 May	5	19.3
M1-2	2013	50	from 6 to 10 mm	1555	0.794 ± 0.04	none	17 May	1	17.8
M1-3	2013	56	from 6 to 10 mm	1487	0.763 ± 0.02	none	6, 17, 18, 30 May	4	18.0
M1-4	2014	86	from 4 to 10 mm	2924	0.807 ± 0.00	none	6, 17, 24, 30 May	4	19.0
