# Supplementary material for: Induction of Terpene Biosynthesis in Berries of Microvine Transformed with VvDXS1 Alleles
Source: Front Plant Sci. 2018 Jan 17;8:2244. doi: 10.3389/fpls.2017.02244 (PMC5776104; doi:10.3389/fpls.2017.02244)
Supplement: Supplementary file 11 [file DataSheet11.PDF]

Table S4. List of primers for gene expression analysis with sybr-green. *Primers pairs were designed in order to specifically amplify (i) the endogenous *VvDXS1* (*VvDXS1_endo*) by using a reverse primer which anneals to the 3'UTR region, (ii) the transgenic *VvDXS1* (*VvDXS1_trans*) by using a reverse primer which anneals to the T35S terminator.

**Gene ID**	**Gene acronym**	**Primer sequence (5'-3')**	**Lenght (bp)**
VIT_05s0020g02130	*VvDXS1*	Fw: CTGTACGCTCTCATTTCCTG<br>Rv: TGATACTCCTCCCTATCGGA	180
	*VvDXS1_endo**	Fw: ATGGCGGGTCTGACACCAT<br>Rv: CAATCTTGACAGAATGAAAAGCTGA	130
	*VvDXS1_trans**	Fw: ATGGCGGGTCTGACACCAT<br>Rv: CCGCGGGATATCAGCACTTT	132
VIT_03s0063g02030	*VvHDR*	Fw: TGAGCACCGAACGGAGAGAG<br>Rv: TTGCTTCCATGGCGATGTCT	125
VIT_12s0057g01200	*VvAACT*	Fw: AGGATGGGCTGTGGGATGTT<br>Rv: CGGAACAATTTCCCATGCAA	170
VIT_02s0025g04580	*VvHMGS*	Fw: GGGGCCAAGGACTTTGTGAC<br>Rv: GCTCCCAGCCTTCTTGCTGT	114
VIT_03s0038g04100	*VvHMGR3*	Fw: ACGTGGGAAATCCGTGGTCT<br>Rv: CCATGGCAGAACCAGTGAGG	125
VIT_09s0002g00100	VvPDS1	Fw: GCCTTGGACATGGCAATGAA<br>Rv: GGGATATGGGCCATCCTGAG	200
VIT_10s0003g03750	VvNCED2	Fw: CGGAGACCAACGAAGTGGTG<br>Rv: TCGATTCACCATGCCTGCTT	191
VIT_12s0134g00020	*VvOciS*	Fw: TCGCTTCGGAAGGAGAAACC<br>Rv: TGGAAGCTCCATGGCATGAT	125
VIT_19s0015g01010	*VvFPPS*	Fw: TGCTGCGAATGATGGGGTGA<br>Rv: ACAATGCGGCGGTGAAGAGG	210

VIT_18s0001g04050	*VvValS*	Fw: GGCATGCTGGGCTTGTATGA<br>Rv: ACCGTGGCCTTGAGGTGAGT	101
